# Application of N-heterocyclic carbene–Cu(I) complexes as catalysts in organic synthesis: a review

**DOI:** 10.3762/bjoc.19.102

**Published:** 2023-09-20

**Authors:** Nosheen Beig, Varsha Goyal, Raj Kumar Bansal

**Affiliations:** 1 Department of Chemistry, The IIS (deemed to be University), Jaipur, 302 020, India

**Keywords:** conjugate addition, [3 + 2] cycloaddition reaction, hydrosilylation reaction, N-heterocyclic carbenes, NHC–Cu complexes, NHC–Cu complexes as catalyst

## Abstract

N-Heterocyclic carbenes (NHCs) are a special type of carbenes in which the carbene carbon atom is part of the nitrogen heterocyclic ring. Due to the simplicity of their synthesis and the modularity of their stereoelectronic properties, NHCs have unquestionably emerged as one of the most fascinating and well-known species in chemical science. The remarkable stability of NHCs can be attributed to both kinetic as well as thermodynamic effects caused by its structural features. NHCs constitute a well-established class of new ligands in organometallic chemistry. Although initially NHCs were regarded as pure σ-donor ligands, later experimental and theoretical studies established the presence of a significant back donation from the d-orbital of the metal to the π* orbital of the NHC. Over the last two decades, NHC–metal complexes have been extensively used as efficient catalysts in different types of organic reactions. Of these, NHC–Cu(I) complexes found prominence for various reasons, such as ease of preparation, possibility of structural diversity, low cost, and versatile applications. This article overviews applications of NHC–Cu(I) complexes as catalysts in organic synthesis over the last 12 years, which include hydrosilylation reactions, conjugate addition, [3 + 2] cycloaddition, A^3^ reaction, boration and hydroboration, N–H and C(sp^2^)–H carboxylation, C(sp^2^)–H alkenylation and allylation, C(sp^2^)–H arylation, C(sp^2^)–H amidation, and C(sp^2^)–H thiolation. Preceding the section of applications, a brief description of the structure of NHCs, nature of NHC–metal bond, and methods of preparation of NHC–Cu complexes is provided.

## Introduction

**Scheme 1:**
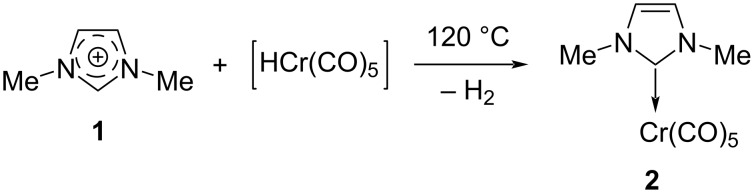
In situ generation of imidazolylidene carbene.

N-Heterocyclic carbenes (NHCs) are a neutral species having the carbene carbon atom as a part of the nitrogen heterocyclic ring. The transient generation of the first NHC can be credited to Öfele [1] who could prepare an imidazolylidene–Cr(VI) complex in 1968 (Scheme 1).

**Scheme 2:**
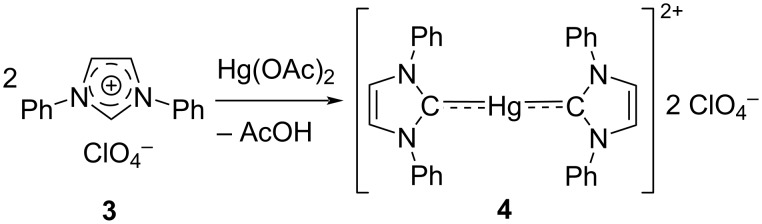
Hg(II) complex of NHC.

Likewise, in the same year, Wanzlick [2] obtained imidazolylidene–Hg(II) complex **4** (Scheme 2).

**Scheme 3:**
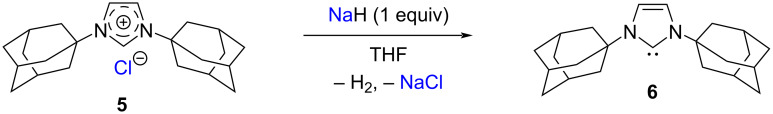
Isolable and bottlable carbene reported by Arduengo [3].

The preparation of an isolable and bottlable NHC, imidazolylidene **6** (Scheme 3) reported by Arduengo et al. [3], turned out to be a watershed in this regard as it initiated intense research activity in this field. Compound **6** is stable in the absence of oxygen and air.

**Scheme 4:**
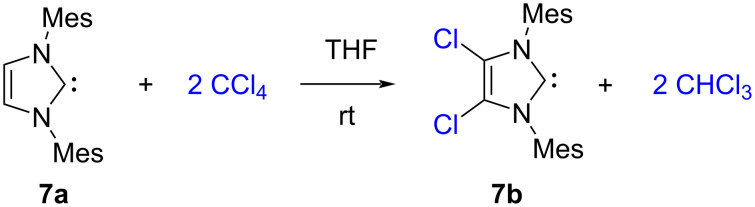
First air-stable carbene synthesized by Arduengo in 1992 [5].

The first air-stable carbene, namely 1,3-dimesityl-4,5-dichloroimidazol-2-ylidene (**7b**) [4] was obtained from the reaction of the carbene 1,3-dimesitylimidazol-2-ylidene (**7a**) with carbon tetrachloride in THF (Scheme 4) [5].

### Structure of N-heterocyclic carbenes

**Figure 1:**
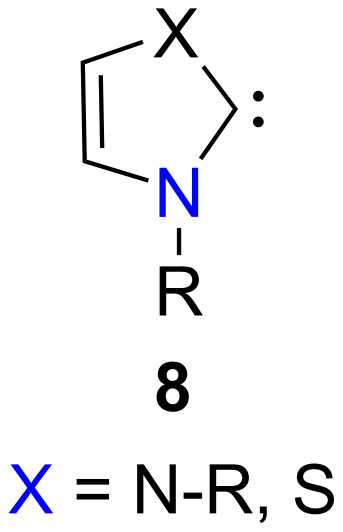
General structure of an NHC.

N-Heterocyclic carbenes contain at least one nitrogen atom and there may be another nitrogen atom or a sulfur atom present in the heterocycle. A general structure of an NHC (**8**) is shown in Figure 1.

**Figure 2:**
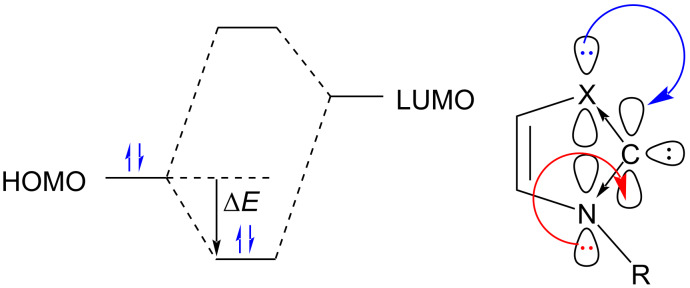
Stabilization of an NHC by donation of the lone pair electrons into the vacant p-orbital (LUMO) at the carbene center.

NHCs having three nitrogen atoms in the five-membered ring, e.g., 1,2,4-triazolylidenes have also been prepared [6]. Both kinetic as well as thermodynamic effects contribute to the remarkable stability of the NHC ring. The presence of bulky substituent groups on the nitrogen atoms adjacent to the carbene center result in kinetic stabilization of the NHC suppressing its dimerization to the corresponding olefin (the Wanzlick equilibrium). On the other hand, thermodynamic stabilization results due to donation of lone pair(s) electrons of the adjacent nitrogen atom(s) or another heteroatom into the vacant p-orbital (LUMO) at the carbene carbon atom (Figure 2). In fact, the donation of lone pair electrons to the carbenic center leads to an ylidic structure which induces a “push–pull” effect thereby enhancing the stability of the NHC. The cyclic structure of the NHCs facilitates this donation and also helps to favor the singlet state by forcing the carbene carbon atom into a bent, more sp^2^-like arrangement.

**Figure 3:**
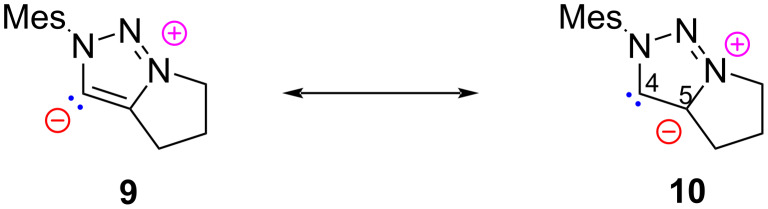
Abnormal NHC reported by Bertrand [8–9].

The thermodynamic stabilization of the NHCs derived from heteroaromatic compounds can be attributed to their partial aromaticity, which was calculated to be around 25 kcal mol^−1^ for model imidazol-2-ylidenes [7]. The ylidic structure confers NHC a nucleophilic character. Bertrand reported a new class of mesoionic carbenes **10** based on a 1,2,3-triazolium scaffold [8–9]. These NHCs, also known as remote or abnormal N-heterocyclic carbenes (aNHCs), bear only a single ﬂanking heteroatom and exist as a zwitterionic structure with no neutral canonical resonance form (Figure 3). In this case, binding takes place via the C4 or C5 position. These NHCs have been found to be stronger electron-donors due to the reduced σ-withdrawal from the carbene carbon atom [10]. As a result of reduced pπ*–*pπ delocalization, these species are more π-accepting in nature [11].

Because of their outstanding electronical characteristics (high σ-donation and π-accepting properties), NHCs have been used extensively in various fields of chemical science over the past 20 years. A number of excellent reviews on different aspects of NHC chemistry has been published during this period [12–14]. NHCs have been widely employed in homogeneous catalysis [12] and as ligands for the preparation of coordination compounds of different metals [13]. The M–NHC bond is relatively stable; as a result, the stability of the resulting metal complexes is enhanced [14]. This has led to the development of a variety of NHC-incorporating metal compounds exhibiting increased activity/selectivity in catalysis [15].

### Nature of the NHC–metal bond

**Figure 4:**
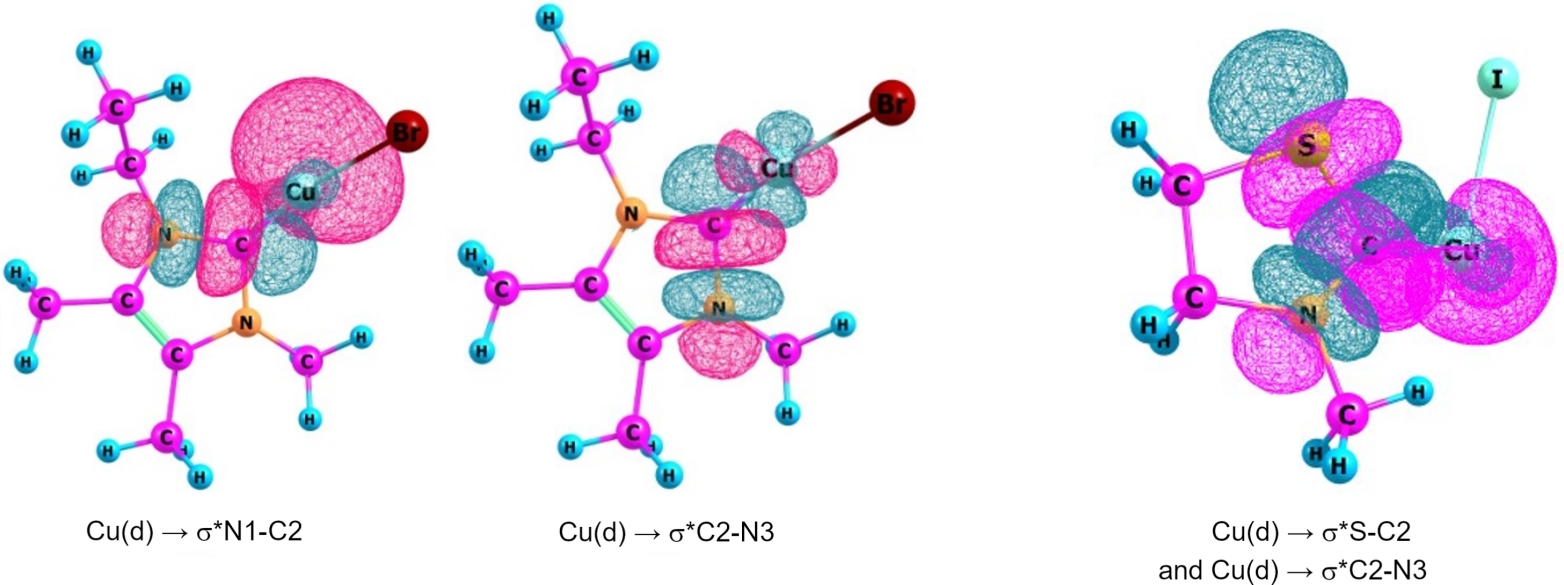
Cu(d) orbital to σ*C-N(NHC) interactions in NHC–CuX complexes computed at the B3LYP/def2-SVP level of theory.

NHCs constitute a well-established class of ligands in organometallic chemistry. After the first synthesis of stable monomeric NHCs, spectroscopic studies promptly revealed their similarity with phosphines. Indeed, both these classes of ligands are σ-donor ligands with low π-backdonating character [16–17]. In the beginning, the NHCs were perceived as σ-donor ligands only. However, subsequent experimental [18] and theoretical [19] studies revealed that a simultaneous back donation from the d-orbital of the metal to the π*-orbital of the NHC occurs in these species, which was also corroborated by energy decomposition analysis (EDA) calculations [20]. We recently carried out the natural bond orbital (NBO) analysis of NHC–CuX complexes revealing the existence of this phenomenon. Such interactions in two NHC–CuX complexes are shown in Figure 4 [21].

**Figure 5:**
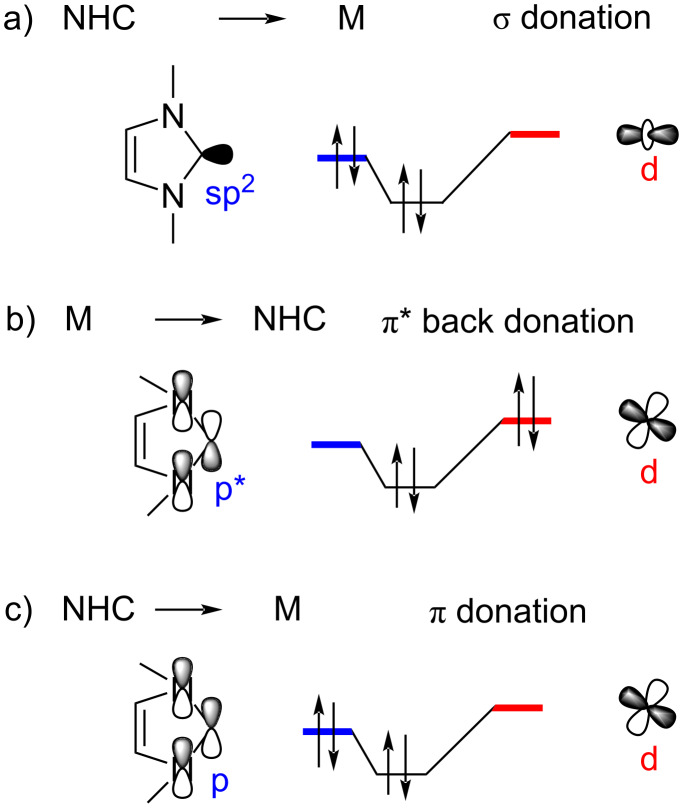
Molecular orbital contributions to the NHC–metal bond.

The molecular orbitals involved in the NHC–metal bonding are shown in Figure 5. There are three electronic interactions noteworthy: part a) represents an NHC-to-metal σ → d donation, part b) shows back bonding from the metal to NHC d → π* donation, and part c) depicts the NHC-to-metal π → d donation.

## Review

In the present review, it is intended to provide an overview of the literature spanning the last 12 years, i.e., from 2010 through 2022 only. Nevertheless, wherever necessary, earlier works may also be included to maintain coherence. Furthermore, in view of the enormous amount of research work done on NHC–metal complexes and their application as catalysts, the present review was restricted to the synthesis and applications of NHC–Cu(I) complexes only.

### 1 Synthesis of NHC–Cu(I) complexes

#### 1.1 Deprotonation of NHC precursors (in situ) with a base

**Scheme 5:**
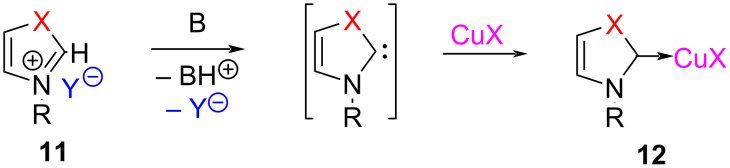
Synthesis of NHC–Cu(I) complexes by deprotonation of NHC precursors with a base.

The *N*-alkylazolium salts **11** upon treatment with a base generate the corresponding NHCs which react with a Cu(I) salt to afford the corresponding NHC–Cu(I) complexes **12** (Scheme 5).

**Scheme 6:**
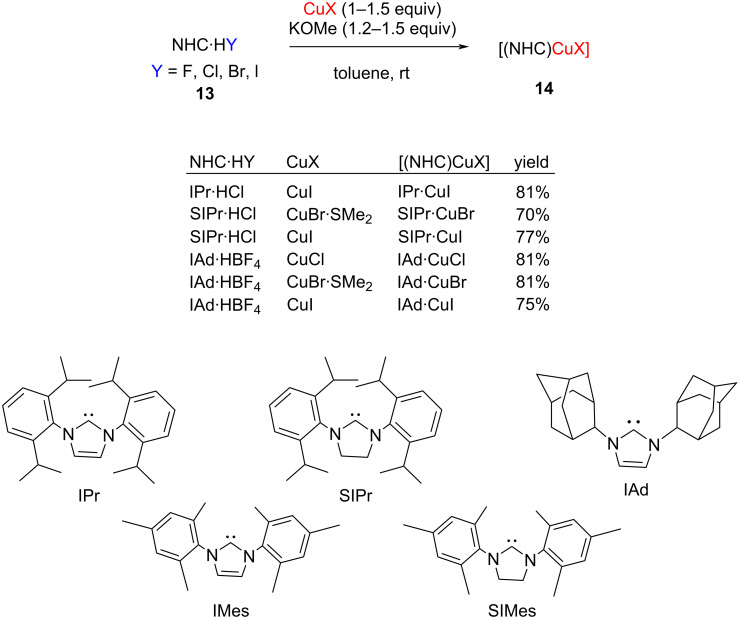
Synthesis of [NHC–CuX] complexes.

A variety of bases, namely metal alkoxides, alkali metal carbonates, *n*-butyllithium, and lithium bis(trimethylsilyl)amide have been employed for this purpose. In 2010, Diez-González et al. synthesized a variety of NHC–Cu(I) complexes **14** from imidazolium salts **13** using alkali alkoxide as the base (Scheme 6) [15].

In the case of IPr·HX, it was found that the use of CuI afforded a significant amount of [(IPr)_2_Cu] complexes even when a large excess of the metal salt was used. Furthermore, the combination of KOMe and toluene afforded a higher yield of [(IPr)CuI] [15]. It may be mentioned here that the complexes [(SIPr)CuX] (X = Cl, Br, I) were also prepared through transmetalation, a method that will be discussed later. In contrast to the aforementioned complexes, [(IMes)CuX] and [(SIMes)CuX] (X = Cl, Br) were best prepared from the corresponding NHC·HCl precursors and copper salts in the presence of NaO*t*-Bu in THF [15]. All attempts to prepare the corresponding iodide-containing compounds resulted in the formation of [(NHC)_2_Cu]^+^ species [15].

**Scheme 7:**
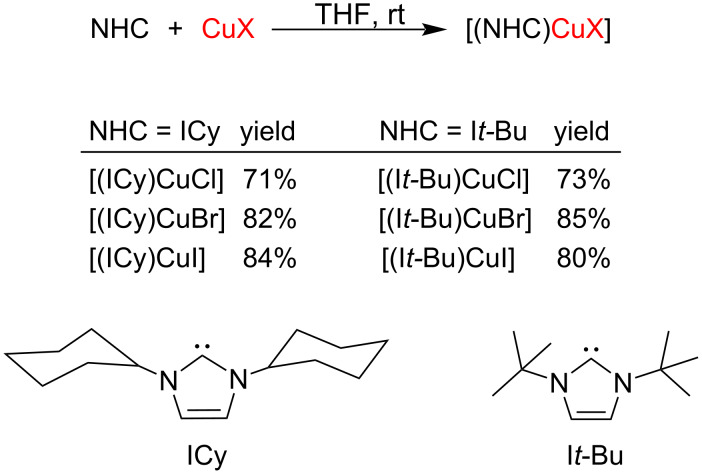
Synthesis of [(ICy)CuX] and [(I*t*-Bu)CuX] complexes.

The copper complexes bearing ICy and I*t*-Bu ligands were found to be sensitive to the excess of base and hence could not be prepared following the above method. Instead, these complexes were best prepared by reacting free NHC with the metal salt (Scheme 7) [15].

**Scheme 8:**
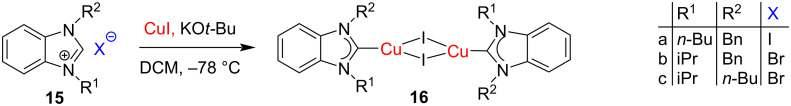
Synthesis of iodido-bridged copper–NHC complexes by deprotonation of benzimidazolium salts reported by Sarkar [22].

In 2011, Sarkar and co-workers [22] also used potassium *tert*-butoxide to obtain NHC–Cu(I) complexes **16** of general composition [(NHC)Cu(µ-I)_2_Cu(NHC)] from benzimidazolium salts **15** in the presence of CuI (Scheme 8).

**Scheme 9:**
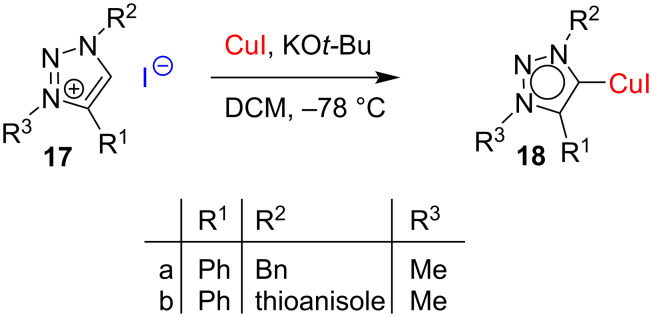
Synthesis of copper complexes by deprotonation of triazolium salts.

In a similar manner, Cu(I) complexes **18** of so-called “abnormal” carbenes [9] or mesoionic carbenes [8], such as 1,2,3-triazol-5-ylidenes have been prepared (Scheme 9). These NHC–Cu(I) complexes have been found to be highly efficient catalysts for the [3 + 2] cycloaddition of azides with alkynes as will be discussed later.

**Scheme 10:**
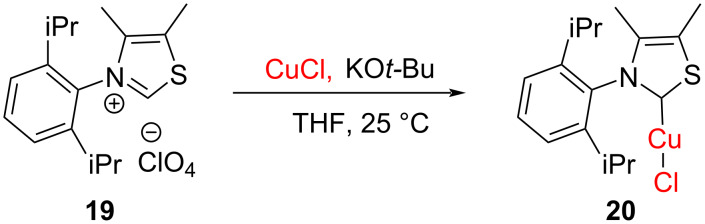
Synthesis of thiazolylidene–Cu(I) complex by deprotonation with KO*t*-Bu.

Jin Zhang et al*.* used the same base, namely KO*t*-Bu in the presence of CuCl to obtain the thiazolylidene–Cu(I) complex **20** (Scheme 10) [23].

**Scheme 11:**
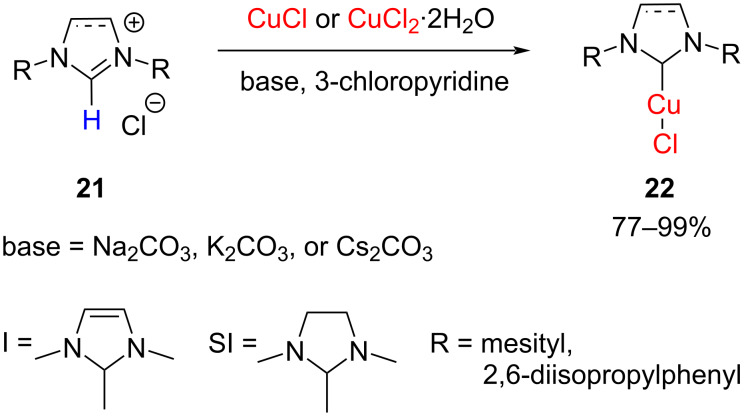
Preparation of NHC–Cu(I) complexes.

In 2012, Zhu and co-workers synthesized NHC–CuCl complexes using alkali carbonates (Na_2_CO_3_, K_2_CO_3_, or Cs_2_CO_3_) in the presence of copper chlorides (Scheme 11). However, this protocol required environmental unfriendly solvents such as 3-chloropyridine and high temperatures. In general, CuCl gave higher yields than CuCl_2_·2H_2_O typically ranging from 70 to 99% [24]. Furthermore, an IMes–CuCl complex was obtained through transmetallation as will be described later.

In 2013, Cazin and co-workers [25] reported the synthesis of [NHC–CuCl] complexes similar to those reported earlier by Diez-González et al. [15], but using K_2_CO_3_ in place of alkali alkoxides under milder conditions. In contrast to the earlier report [15], under these conditions, formation of [(NHC)_2_Cu]^+^ complexes was not observed.

**Scheme 12:**
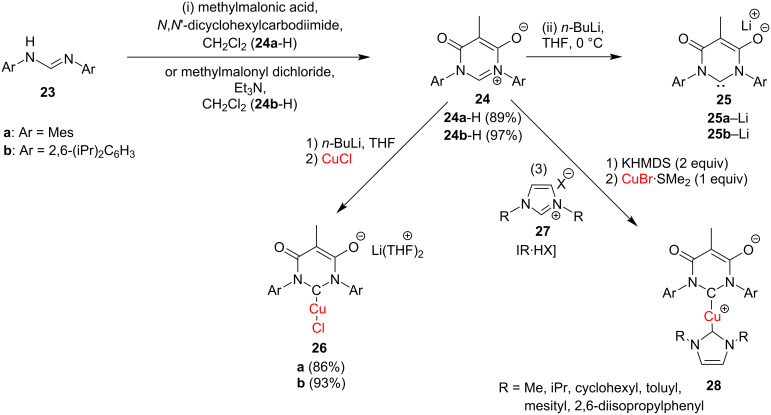
Synthesis of methylmalonic acid-derived anionic [(**26a**,**b**)CuCl]Li(THF)_2_ and zwitterionic (**28**) heteroleptic Cu(I) complexes.

In the same year, César et al*.* [26] reported in situ-generated malonate-derived anionic carbenes which reacted with CuCl to afford the anionic [(maloNHC)CuCl]Li complexes. Furthermore, zwitterionic heteroleptic Cu(I) complexes combining the malonic acid-derived anionic NHC and a neutral imidazol-2-ylidene were also obtained in a very selective manner (Scheme 12). As discussed later, many of these complexes were employed as catalysts.

**Scheme 13:**
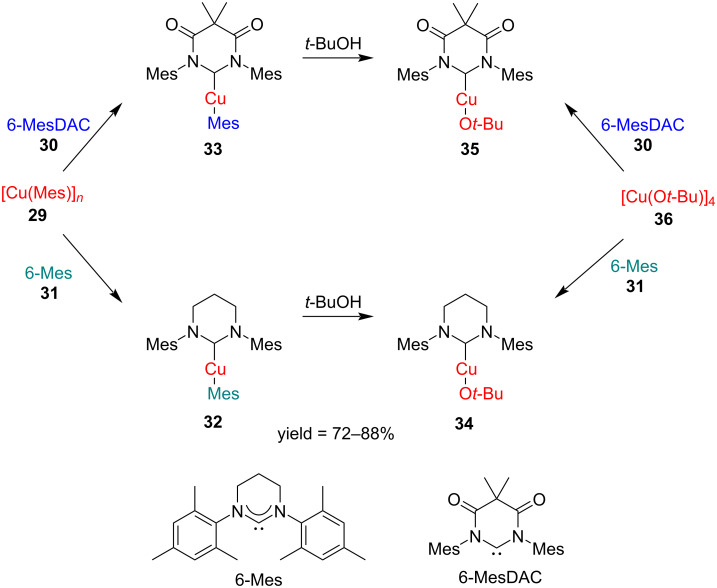
Synthesis of diaminocarbene and diamidocarbene (DAC)–Cu(I) complexes.

In 2015, Collins et al*.* [27] compared the stability and reactivity of diaminocarbene complex **32** and diamidocarbene (DAC) complex **33**. The mononuclear N-heterocyclic carbene (NHC)–copper alkoxide complexes [(6-NHC)CuO*t*-Bu] (6-NHC = 6-MesDAC (**30**), 6-Mes (**31**)) were prepared by the addition of free carbenes to the tetrameric *tert*-butoxide precursor [Cu(O*t*-Bu)]_4_, or by protonolysis of [(6-NHC)CuMes] with *t*-BuOH. It was found that the diaminocarbene derivative was more thermally stable whereas the diamido derivative underwent both thermal and hydrolytic ring-opening reactions (Scheme 13).

**Scheme 14:**
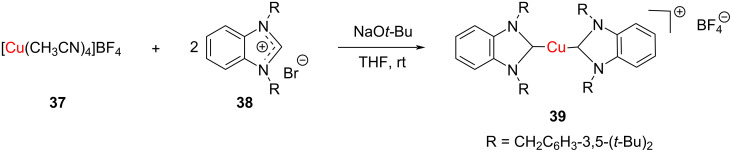
Synthesis of the cationic (NHC)_2_Cu(I) complex **39** from benzimidazolium salts **38** with tetrakis(acetonitrile)copper(I) hexafluorophosphate reported by Demir Atli [28].

Demir Atli and co-worker, in 2018, synthesized a cationic (NHC)_2_Cu(I) complex **39** in very good yield by reacting [Cu(CH_3_CN)_4_]BF_4_ with 2 equiv of benzimidazolium salt **38** in the presence of NaO*t*-Bu (Scheme 14) [28]. The catalytic activity of the complex **39** was studied in the [3 + 2] cycloaddition of azides with alkynes [28].

**Scheme 15:**
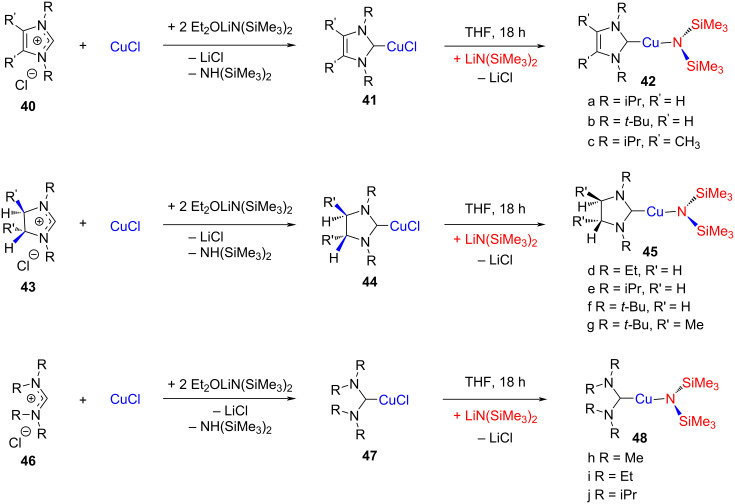
Synthesis of NHC and ADC (acyclic diamino carbenes) Cu(I) hexamethyldisilazide complexes reported by Coyle et al. [29].

Coyle et al. reported the synthesis of a series of NHC and ADC (acyclic diaminocarbenes) Cu(I) hexamethyldisilazide complexes by using lithium hexamethyldisilazide as a base in 2017 (Scheme 15) [29]. The initially formed NHC–CuCl complexes **41**, **44**, and **47** reacted with another molecule of LiN(SiMe_3_)_2_ to undergo nucleophilic substitution of Cl by a bis(trisilyl)amino group to furnish the NHC–CuN(SiMe_3_)_2_ complexes **42**, **45**, and **48**, respectively. All synthesized complexes were characterized by TGA to explain their thermal behavior. The impact of the *N*-allyl substituent and backbone character on volatility and thermal stability of the copper amides were investigated and it was found that the saturated copper–carbene complexes exhibit a higher thermal stability as compared to the unsaturated copper–carbene complexes.

**Scheme 16:**
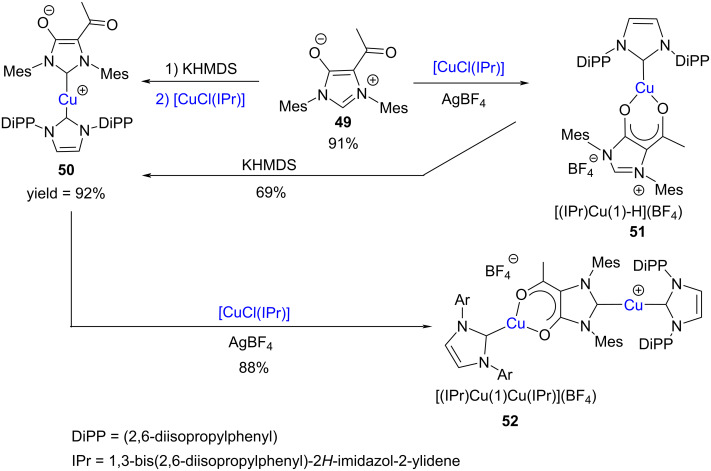
Synthesis of NHC–copper(I) complexes using an acetylacetonate-functionalized imidazolium zwitterion as a bidentate ligand.

In another interesting research paper, César et al*.*, in 2015, reported the synthesis of the mesoionic 5-acetylimidazolium-4-olate **49** which served as a precursor for an anionic hybrid NHC, “IMes-acac” consisting of fused diaminocarbene and acetylacetonato units. The latter afforded a series of representative Cu(I) complexes through bidentate coordination (Scheme 16) [30].

#### 1.2 Deprotonation of NHC-precursors with Cu_2_O

**Scheme 17:**
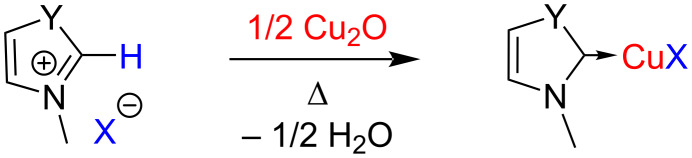
Synthesis of NHC–Cu(I) complexes through deprotonation of azolium salts with Cu_2_O.

Another important and facile method involves heating of the NHC-precursor, i.e. an azolium salt with cuprous oxide using a solvent or under microwave conditions (Scheme 17).

**Scheme 18:**
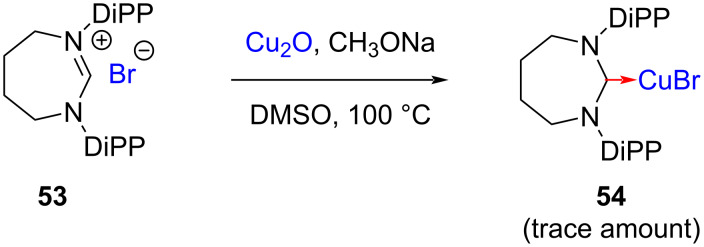
Synthesis of NHC–CuBr complex through deprotonation with Cu_2_O reported by Kolychev [31].

Kolychev in 2009 attempted the preparation of NHC–Cu(I) complexes via deprotonation by heating of amidinium salt **53** [(7-Dipp)H][Br] with Cu_2_O in the presence of sodium acetate in DMSO. However, the Cu(I) complex **54** was obtained in trace amounts only (Scheme 18) [31]. This complex could be obtained in good yield through transmetallation of the corresponding Ag complex as discussed later in Scheme 24.

**Scheme 19:**
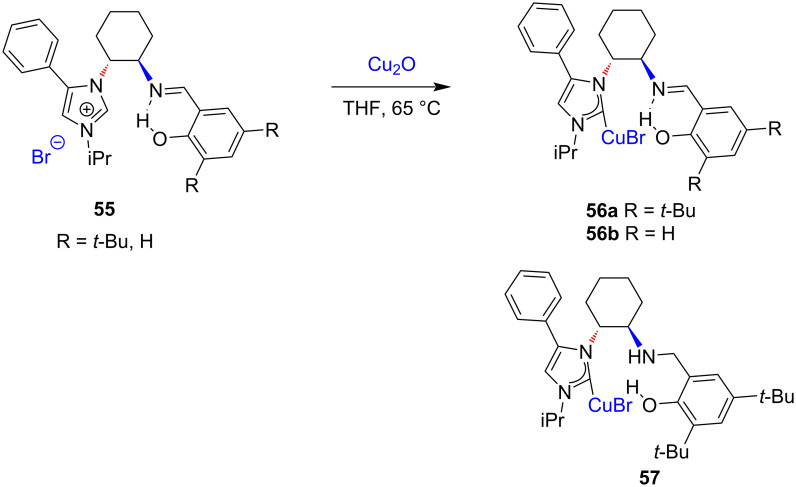
Synthesis of chiral NHC–CuBr complexes from phenoxyimine-imidazolium salts reported by Douthwaite and co-workers [32].

Douthwaite and co-workers reported the synthesis of Cu(I) bromide complexes **56a**,**b** from the reaction of chiral NHC precursors based on a phenoxyimine-imidazolium motif with Cu_2_O in THF in >90% yield. The reaction solvent was found to be important; in dichloromethane, interactable products were formed in contrast to the excellent yields obtained in THF. Furthermore, the NHC–CuBr complex **57** was obtained through reduction of the phenoxyimine-imidazolium bromide **55a** (R = *t*-Bu) with NaBH_4_ followed by successive alkylation with iPrBr and reaction with Cu_2_O (Scheme 19) [32]. The use of the synthesized complexes **56a** and **57** as precatalysts for the 1,4-conjugate addition to enones and the aziridination of alkenes was also investigated [32].

**Scheme 20:**
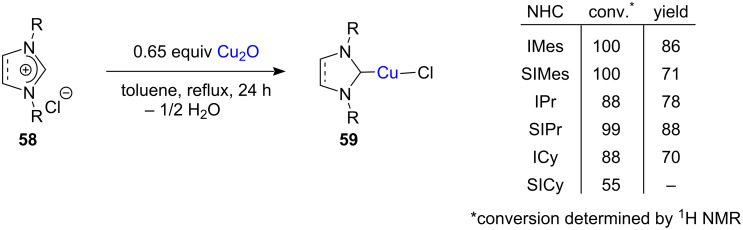
Preparation of linear neutral NHC–CuCl complexes through the use of Cu_2_O. For abbreviations, please see Scheme 6 and Scheme 7.

Cazin and co-workers reported the preparation of linear neutral NHC–CuCl complexes from the reaction of imidazolium and imidazolinium chlorides with Cu_2_O. Initially, dichloromethane was used as the solvent. However, except in the case of IMes and SIPr, the yields of the products were poor. Then, a non-chlorinated solvent such as toluene was used which furnished the complexes in moderate to excellent yields (Scheme 20). The use of water as solvent was found to be viable in the case of the NHCs bearing aryl substituent groups. However, the reaction of alkyl *N*,*N*’-disubstituted salts, e.g., dicyclohexyl-substituted salt (ICy, SICy), to form the Cu complexes seemed somehow to be impeded [33]. Some of these complexes were used as NHC transfer reagents to obtain Au(I) and Pd(II) complexes through transmetallation [34].

**Scheme 21:**
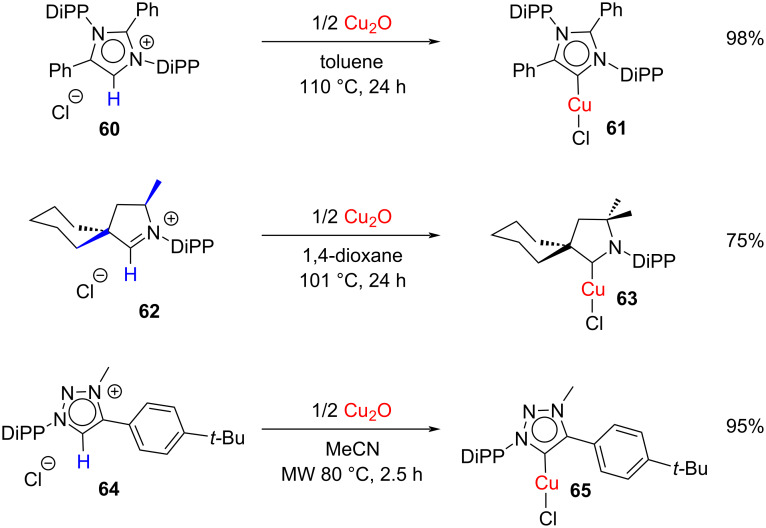
Synthesis of abnormal-NHC–copper(I) complexes by Bertrand, Cazin and co-workers [35].

In another interesting paper, Bertrand, Cazin and co-workers reported the synthesis of Cu(I) complexes **61**, **63**, and **65** of the so-called ‘abnormal’ NHCs (Scheme 21) [35]. Thus, the conventional heating method as well as microwave irradiation methods were employed. The structures of the synthesized complexes were confirmed by X-ray analysis which showed that all complexes adopted a slightly distorted linear geometry. Furthermore, abnormal-NHC and triazolylidene-based Cu(I) complexes exhibited an outstanding catalytic property towards the [3 + 2] cycloaddition of alkynes with azides.

**Scheme 22:**
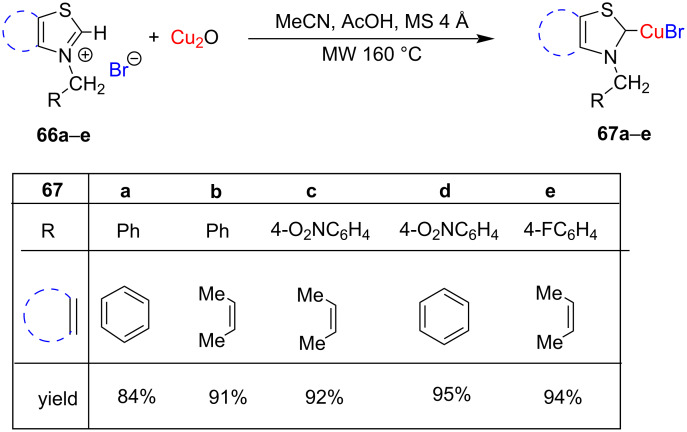
Microwave**-**assisted synthesis of thiazolylidene/benzothiazolylidene–CuBr complexes by Bansal and co-workers [36].

Recently, we have reported the synthesis of NHC thiazolylidene–CuBr complexes **67a–e** through microwave-assisted deprotonation of *N*-alkylthiazolium/benzothiazolium bromides with Cu_2_O in the presence of MeCN/AcOH and molecular sieves. The complexes were obtained in high (84–96%) yields (Scheme 22) [36].

#### 1.3 Transmetallation

The free NHC route, although still employed for the preparation of numerous NHC–Cu complexes, requires the use of strong bases and rigorous inert conditions. However, an alternative method called transmetallation method has also been employed for the preparation of NHC–Cu(I) complexes by using NHC transfer reagents. For this purpose, NHC–Ag(I) complexes are frequently used to prepare NHC complexes of late transition metals [37].

**Scheme 23:**
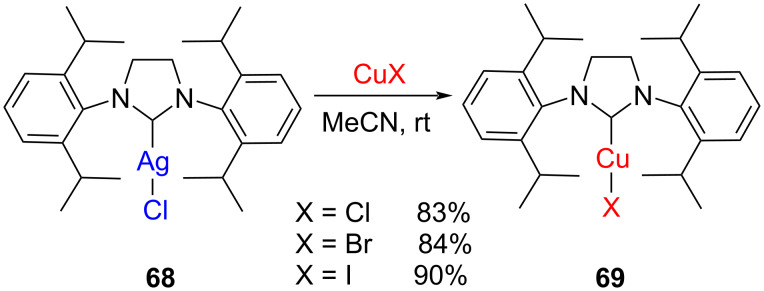
Synthesis of NHC–CuX complexes through transmetallation.

As mentioned earlier, Diez-González et al*.* prepared some NHC–Cu(I) complexes, such as **69** through transmetallation by reacting [(SIPr)AgCl] **68** with the corresponding copper salt at rt (Scheme 23). However, mixtures of mono- and bisNHC complexes were systematically obtained from [(IMes)AgCl], [(SIMes)AgCl], and [(ICy)AgCl] [15].

**Scheme 24:**
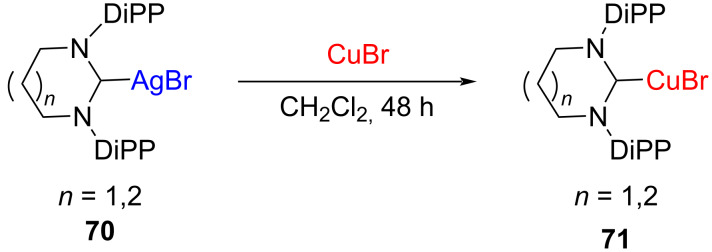
Preparation of six- or seven-membered NHC–Cu(I) complexes through transmetalation from Ag(I) complexes reported by Kolychev et al. [31].

Kolychev obtained NHC–Cu(I) complexes **71** in high yields through transmetallation by reacting an equimolar amount of [(6-Dipp)AgBr] (**70**, *n* = 1) or [(7-Dipp)AgBr] (**70**, *n* = 2) with copper(I) bromide in CH_2_Cl_2_ (Scheme 24) [31].

**Scheme 25:**
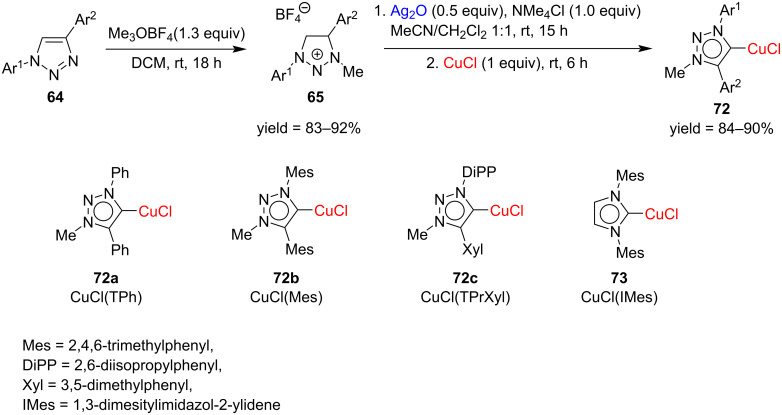
Synthesis of 1,2,3-triazolylidene–CuCl complexes through transmetallation of Ag(I) complexes generated in situ.

Nakamura et al*.* synthesized for the first time a copper complex with a 1,2,3-triazole carbene ligand in 2011. Complexes of copper with 1,4-diphenyl-, 1,4-dimesityl-, and 1-(2,6-diisopropylphenyl)-4-(3,5-xylyl)-1,2,3-triazol-5-ylidene were prepared through consecutive treatment of the corresponding azolium salts with silver oxide and copper chloride (Scheme 25). The X-ray structure of one of the complexes, [(TPrXyl)CuCl], revealed that the NHC–Cu–Cl bond angle is 177.8°, indicating almost linearity. These synthesized complexes were also used as efficient catalysts in the click reaction of azides with alkynes at rt [38].

**Scheme 26:**
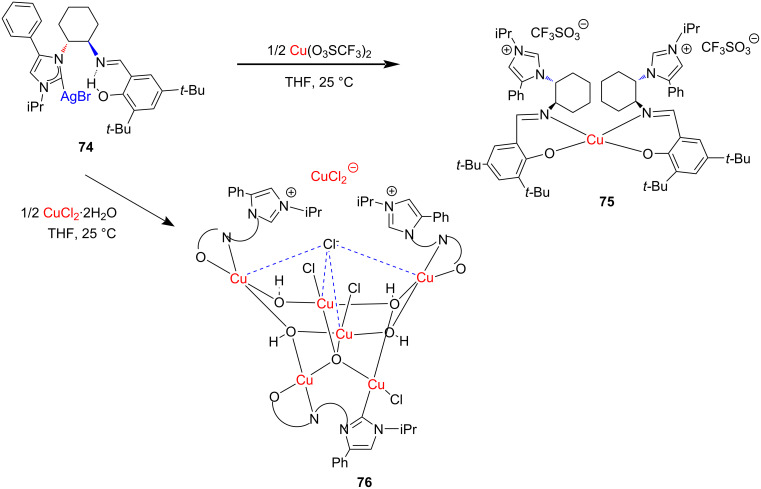
Synthesis of NHC–copper complexes having both Cu(I) and Cu(II) units through transmetalation reported by Douthwaite and co-workers [32].

As discussed earlier, Douthwaite and co-workers obtained Cu(I) bromide complexes **56a**,**b** through deprotonation of the NHC precursor with Cu_2_O (Scheme 19). During workup of complexes **56a** and **56b**, two more complexes, **75** and **76**, were isolated respectively, incorporating NHC–Cu(I) bromide and Cu(II) phenoxymine coordination. These complexes could be independently prepared through transmetallation by reacting NHC–AgBr **74** with copper(II) precursors Cu(SO_3_CF_3_)_2_ or CuCl_2_·2H_2_O (Scheme 26) [32].

**Scheme 27:**
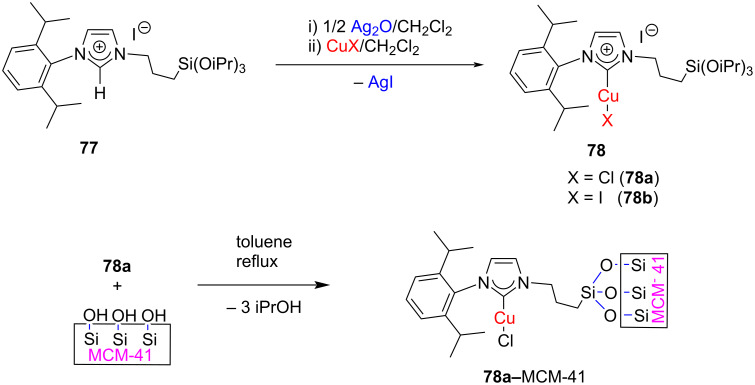
Synthesis of new [(IPr(CH_2_)_3_Si(OiPr)_3_)CuX] complexes and anchoring on MCM-41.

Oro and co-workers synthesized new NHC–CuX complexes **78a**,**b** having a triisopropoxy(propyl)silyl ((-CH_2_)_3_Si(OiPr)_3_) substituent on the imidazole ring through in situ transmetallation. One of these complexes, **78a**, was successfully anchored on mesoporous silica MCM-41 to afford a new heterogeneous catalyst (Scheme 27). Both compounds were subsequently used as catalysts for hydrosilylation and [3 + 2] cycloaddition discussed later [39].

#### 1.4 By ligand displacement

**Scheme 28:**
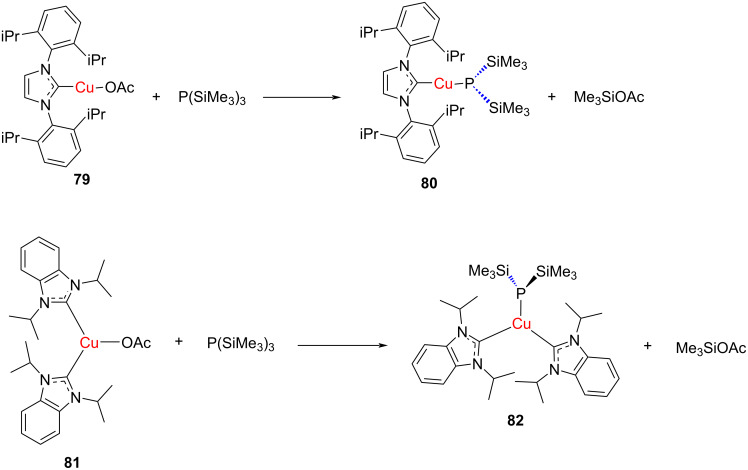
Synthesis of bis(trimethylsilyl)phosphide–Cu(I)–NHC complexes through ligand displacement.

Corrigan and co-worker stabilized homoleptic copper- and silver bis(trimethylsilyl)phosphido compounds [M_6_{P(SiMe_3_)_2_}_6_] (M = Cu, Ag) through their coordination with NHC ligands. For this purpose, they used 1,3-diisopropylbenzimidazol-2-ylidene (iPr_2_-bimy) and 1,3-bis(2,6-diisopropylphenyl)imidazol-2-ylidene (IPr) (Scheme 28) [40]. The structures of all the synthesized complexes were confirmed by X-ray crystallography. A similar strategy was followed for stabilizing copper- and silver *tert-*butylthiolate clusters [41].

**Scheme 29:**
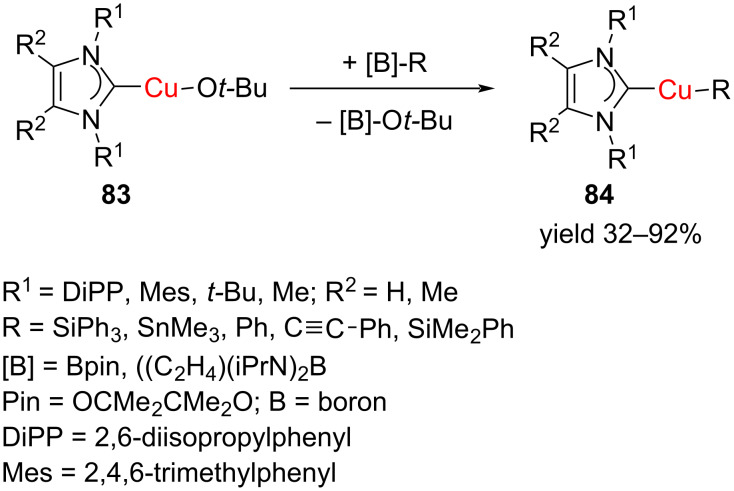
Synthesis of silyl- and stannyl [(NHC)Cu−ER_3_] complexes.

Following a similar strategy, a series of silyl and stannyl-substituted NHC–Cu(I) complexes were prepared through the reaction of [(NHC)Cu–O*t*-Bu] with the respective silyl and stannylboranes. In addition, it was possible to obtain phenyl and alkynyl complexes [(NHC)Cu–R] (R = Ph, C
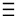
C-Ph) in this manner (Scheme 29) [42]. The X-ray studies revealed two major structure types of the compounds [(NHC)Cu-ER_3_] in the solid state. In the case of bulky NHC ligands, such as IDipp, IMes, and I*t*-Bu, the CuI complexes with ER_3_ = SiMe_2_Ph, SiPh_3_, SnMe_3_ as well as for Ph and C
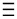
C-Ph, have monomeric nearly linear structures. However, with the methyl-substituted NHC (Me_2_IMe), dimeric silyl complexes were found to have a butterfly shaped Cu_2_Si_2_ core with μ-silyl ligands bridging the two Cu ions. A structurally related trimeric complex was also obtained for the trimethylstannyl complex. An ultrashort Cu···Cu distance of ca. 2.3 Å was detected in these complexes.

**Scheme 30:**
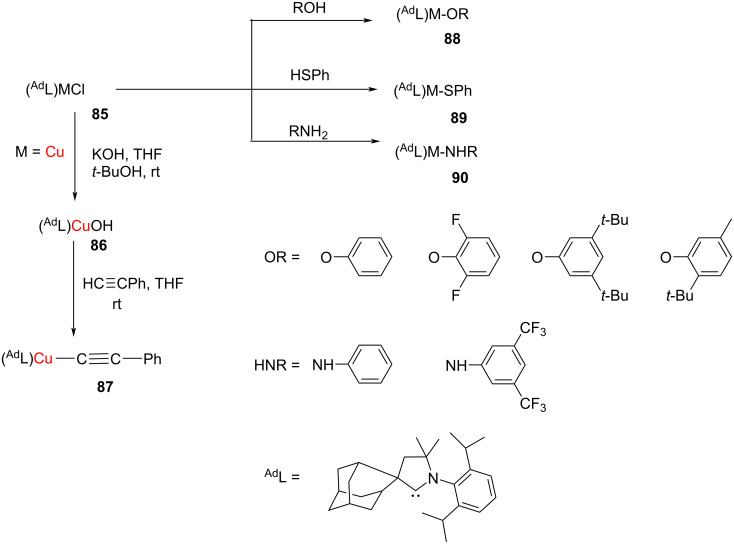
Synthesis of amido-, phenolato-, thiophenolato–Cu(NHC) complexes.

In 2017, Romanov et al*.* synthesized a series of amido and (phenolato)Cu–NHC complexes from the reaction of (^Ad^L)MCl (M = Cu, Au) with phenols, thiophenols, or aromatic amines in the presence of NaO*t*-Bu at rt in anhydrous THF (Scheme 30). The complexes were obtained in high yields (>95%). The copper complexes were air and moisture-stable as solids but decomposed in chlorinated solvents after several days. It may be mentioned that Cu(I) halide and pseudohalide complexes with NHCs ^Me2^L, ^Et2^L and ^Ad^L were prepared from direct reaction of the respective ligand with the copper salts in THF [43].

**Scheme 31:**
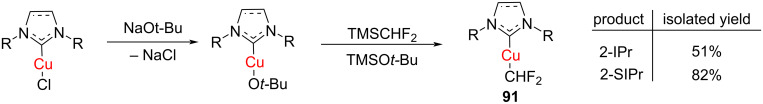
Synthesis of first isolable NHC–Cu–difluoromethyl complexes reported by Sanford et al. [44].

In the same year, Sanford and co-workers [44] synthesized the first isolable NHC–Cu(I)–difluoromethyl complexes **91** (Scheme 31). Owing to the low stability of [Cu(CHF_2_)] species, larger/bulky ligands like IPr, SiPr, etc. were used to obtain stable monomeric copper complexes. The X-ray structure shows a nearly linear C–Cu–CHF_2_ bond angle of 178°.

**Scheme 32:**
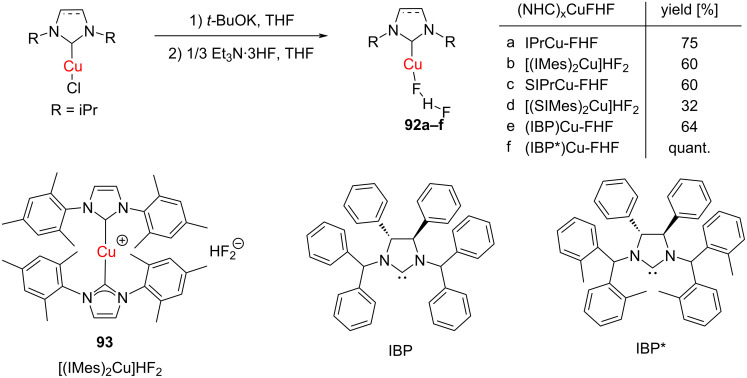
Synthesis of NHC–Cu(I)–bifluoride complexes reported by Riant, Leyssens and co-workers [45].

Riant, Leyssens and co-workers obtained NHC–Cu(I)–bifluoride complexes **92** by reacting (IPr)CuCl successively with KO*t*-Bu and NEt_3_·3HF under inert atmosphere (Scheme 32) [45]. In place of KO*t*-Bu and NEt_3_·3HF, AgHF_2_ could also be used. The complexes were found to be air-stable in the solid state and moderately stable in solution. They were unambiguously characterized on the basis of NMR studies including ^19^F NMR and X-ray crystal analysis. With the IMes ligand, an air-stable bis(NHC)Cu(I) complex [(IMes)_2_Cu]^+^FHF^−^ (**91**) was also obtained. These complexes exhibit an interesting catalytic activity that will be described later.

### 2 Application as catalysts

**Scheme 33:**
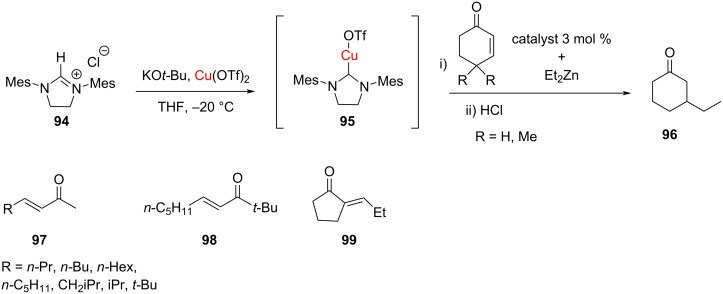
Conjugate addition of Et_2_Zn to enones catalyzed by an NHC–Cu(I) complex reported by Woodward in 2001 [46].

In 2001, Woodward, for the first time, reported an NHC–Cu-mediated catalysis in the conjugate addition of diethylzinc to enones (Scheme 33) [46].

Later in 2003, Buchwald, Sadighi and Jurkauskas [47] succeeded in the application of [(IPr)CuCl] as NHC–Cu(I) complex to catalyze the conjugate reduction of α,β-unsaturated carbonyl compounds. In the decade following these initial reports, the field has blossomed and NHC–Cu(I) complexes have been employed as catalysts in a variety of organic reactions. In this section, a brief review on the use of NHC–Cu(I) complexes as catalysts in organic synthesis is presented.

#### 2.1 Hydrosilylation reactions

**Scheme 34:**
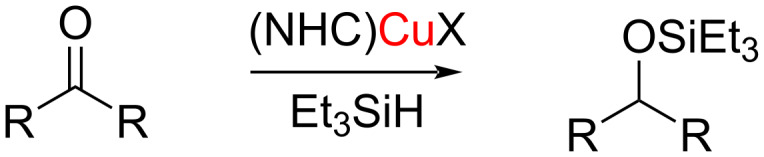
Hydrosilylation of a carbonyl group.

Hydrosilylation reactions involve the addition of a silicon–hydrogen (Si–H) moiety across a carbon–carbon or carbon–heteroatom double bond, resulting in the formation of a new carbon–silicon (C–Si) or heteroatom–Si (X–Si) bond (Scheme 34). This reaction is widely used in the synthesis of organosilicon compounds and in the modification of surfaces with silicon-containing molecules.

NHC–Cu complexes have been found to be highly efficient catalysts in this transformation. Nolan and co-workers reported the catalytic activity of [Cu(IPr)Cl] in the hydrosilylation of carbonyl compounds to form silyl ethers in high yield [48–49].

**Scheme 35:**
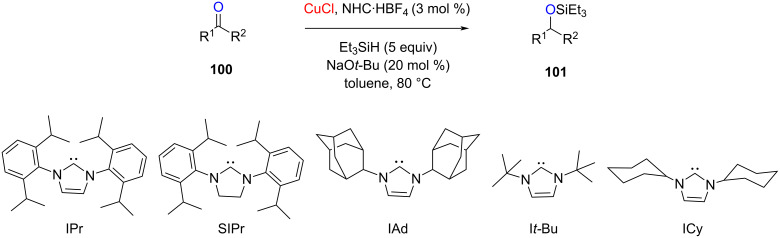
NHC–Cu(I)-catalyzed hydrosilylation of ketones reported by Nolan et al. [48–49].

A series of bis-NHC–copper complexes was synthesized and the compounds were explored as catalysts for the hydrosilylation of ketones. Besides the type of the ligand in the NHC–Cu(I) complex, the counter anions (BF_4_ and PF_6_) also influenced the catalytic activity. Furthermore, the cationic complexes were found to be more efficient than the neutral analogues under similar conditions. The hydrosilylation of aryl, alkyl, and cyclic ketones could be accomplished with excellent yields (Scheme 35) [48].

**Scheme 36:**
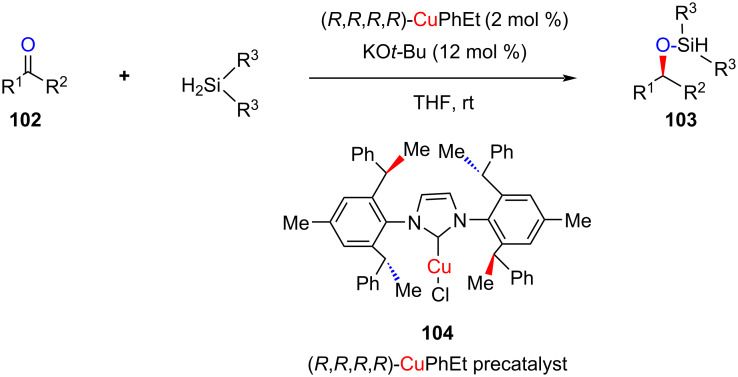
Application of chiral NHC–CuCl complex **104** for the enantioselective hydrosilylation of ketones.

In 2011, Gawley and co-worker reported an excellent reactivity and enantioselectivity of a *C*_2_-symmetric NHC–Cu(I) complex for the catalytic hydrosilylation of a variety of carbonyl compounds, including diaryl- and dialkyl ketones and prochiral ketones, respectively (Scheme 36) [50].

Riant, Leyssens and co-workers investigated the mechanism of the hydrosilylation reaction of ketones using DFT, in situ FTIR, NMR, and kinetic methods [51–52].

**Scheme 37:**
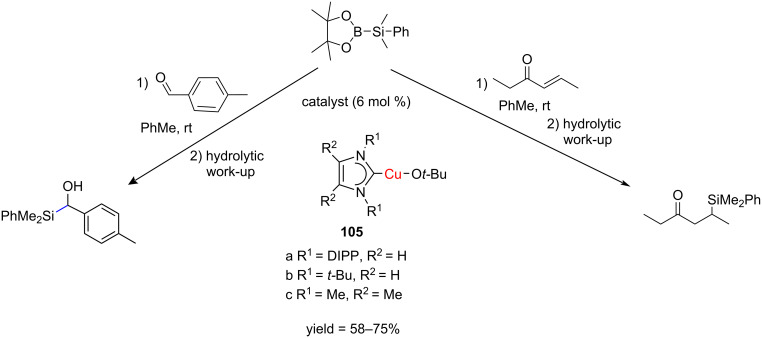
Hydrosilylation reactions catalyzed by NHC–Cu(O*t*-Bu) complexes.

The catalytic characteristics of sterically less-encumbered NHC–CuSiMe_2_Ph complexes for the silylation of aldehydes and unsaturated ketones with silylboranes were investigated in a case study. The complexes (I*t*-Bu)CuO*t*-Bu (**105b**) and (Me_2_IMe)CuO*t*-Bu (**105c**) were found to be effective (pre)catalysts for the 1,2-silylation of tolualdehyde and the 1,4-silylation of hex-3-ene-4-one. An efficient conversion was observed in both cases and the silylated products were isolated in good yields (Scheme 37) [42].

**Scheme 38:**
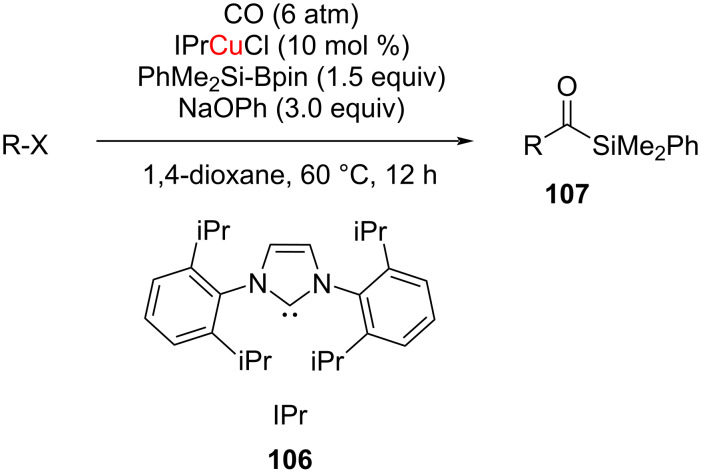
NHC–CuCl catalyzed carbonylative silylation of alkyl halides.

Recently, in 2021, Cheng and Mankad reviewed NHC–Cu(I)-catalyzed carbonylative coupling reactions including the carbonylative silylation of alkyl halides (Scheme 38) [53]. Carbonylative silylation of unactivated alkyl halides was achieved by using the commercially available Si nucleophile PhMe_2_Si-Bpin in the presence of IPrCuCl complex **106** as catalyst (Scheme 38). This allowed to obtain alkyl-substituted acylsilanes **107** in high yields from primary, secondary, and tertiary alkyl halides. The mechanistic investigation revealed the generation of a silyl–copper intermediate which activates the alkyl halides by a single electron transfer to form alkyl radical intermediates [54]. It was suggested that substituting B_2_pin_2_ for PhMe_2_Si-Bpin would allow to synthesize acylboron compounds [54].

As mentioned earlier, Oro and co-workers [39] used 5 mol % of complex **78a** (Scheme 27) for the homogeneous hydrosilylation of acetophenone with HSiEt_3_ in the presence of KO*t*-Bu. As indicated by ^1^H NMR spectroscopy, the conversion was complete in 18 h. Unexpectedly, no reaction occurred when complex **78** anchored on mesoporous silica **78a-MCM-41** was used as heterogeneous catalyst.

#### 2.2 Conjugate addition

**Scheme 39:**
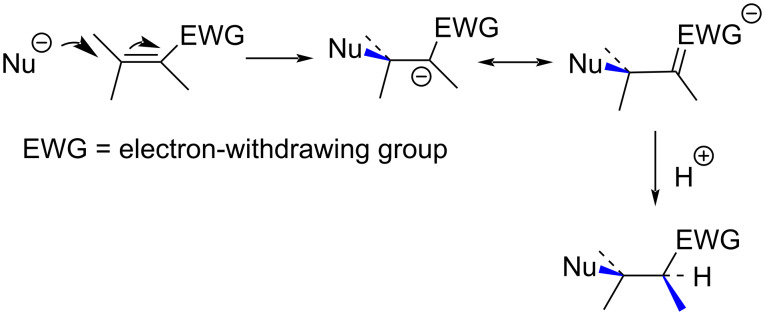
Nucleophilic conjugate addition to an activated C=C bond.

The conjugate addition is a reaction in which a nucleophile attacks at the β-position of an activated C=C moiety to give an addition product (Scheme 39).

**Figure 6:**
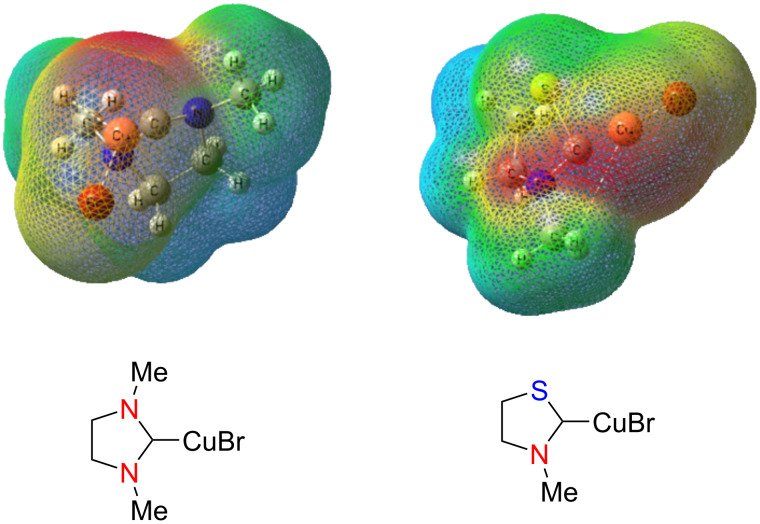
Molecular electrostatic potential maps (MESP) of two NHC–CuX complexes computed at the B3LYP/def2-SVP level of theory.

As mentioned earlier, we recently reported the results of the theoretical investigation of different classes of NHCs and their NHC–Cu(I) complexes at the DFT level [21]. It was shown that in the NHC–Cu(I) complexes, the negative charge density is located mainly in the CuX region. The molecular electrostatic potential (MESP) maps of two such complexes are shown in Figure 6.

It is noteworthy that the intensity of the red color indicative of the negative charge density is maximum around the Cu atom conferring a nucleophilic character on it. Thus, the reaction is initiated by the nucleophilic attack of the NHC–Cu(I) complex at the β-carbon atom of the activated C=C bond thereby catalyzing the reaction.

The NHC–Cu(I) complexes have been found to be effective catalysts for conjugate addition reactions because they stabilize the intermediate species involved in the reaction, which can otherwise be highly reactive and difficult to isolate. The NHC–Cu complex also helps to control the regioselectivity and stereoselectivity of the reaction, which are of high importance in organic synthesis. Overall, the use of NHC–Cu complexes as catalysts for conjugate addition reactions offers a highly efficient and selective method for the synthesis of a wide range of organic compounds [55–56]. Organometallic reagents, such as organolithium, organomagnesium, and organozinc reagents are commonly used in conjugate addition reactions.

**2.2.1 Reaction with Grignard reagents:** Organomagnesium reagents, such as Grignard reagents, are commonly used in conjugate addition reactions. In the presence of Grignard reagents, the NHC-precursor salts do not require an addition of base as the Grignard reagent itself performs this role.

**Scheme 40:**
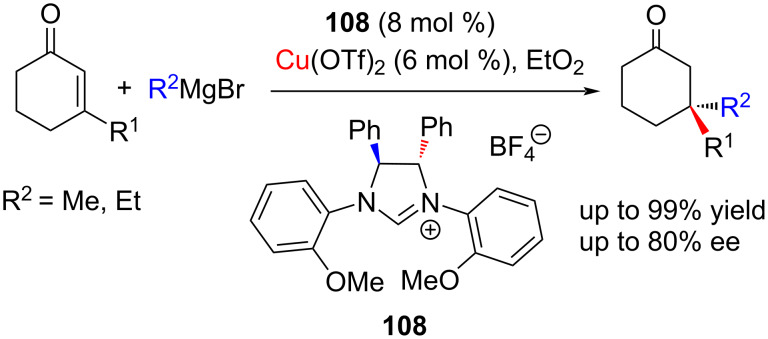
Conjugate addition of Grignard reagents to 3-alkyl-substituted cyclohexenones catalyzed by a chiral in situ-generated NHC–Cu complex.

In this way, Tomioka and co-workers [57] were able to achieve excellent regio- and enantioselectivity, using NHC–Cu(I) complexes generated in situ from chiral imidazolium salts containing possible chelating functional group(s). For example, the conjugate addition of Grignard reagents to 3-methyl- and 3-ethylcyclohexenones in the presence of the *C*_2_-symmetric chiral NHC–copper complex catalyst generated in situ from imidazolium tetrafluoroborate salt **108** and Cu(II) triflate afforded the products in up to 99% yield and up to 80% ee (Scheme 40). On using other NHC precursors, byproducts were formed resulting in lowering of the yield and ee.

**Scheme 41:**
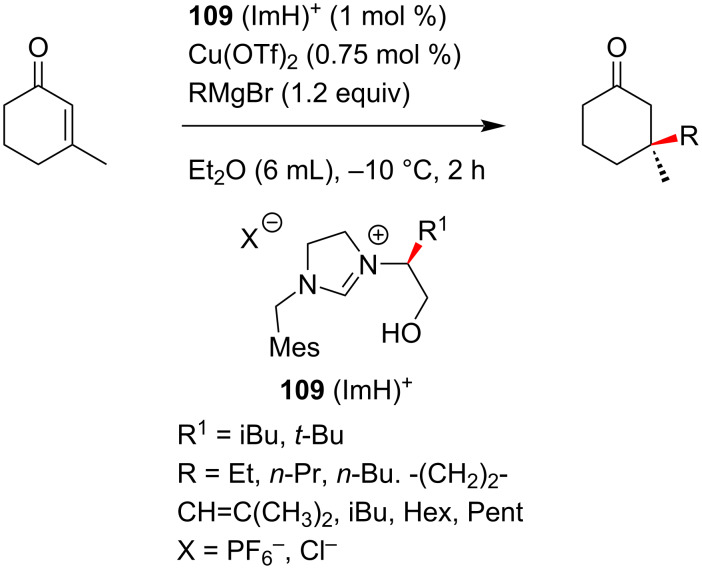
NHC–copper complex-catalyzed conjugate addition of Grignard reagent to 3-substituted hexenone reported by Alexakis and co-workers [58–59].

Alexakis and co-workers [58–59] also followed the same strategy exploring different NHC precursors and a variety of Grignard reagents. The products were obtained in high yields and ee (Scheme 41).

In this context, it was found that higher dilution with a lower catalyst loading (0.75 mol % Cu(OTf)_2_ and 1 mol % NHC) resulted in an increase of ee to 93%. The use of imidazolium salt **109** (R^1^ = iBu) resulted in the highest level of stereoinduction for the conjugate addition of EtMgBr to 3-methylcyclohexenone.

**Scheme 42:**
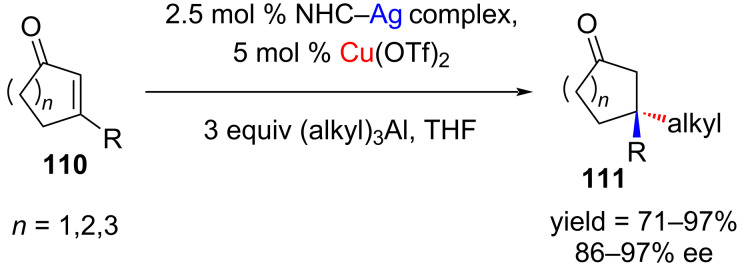
Conjugate addition or organoaluminum reagents to β-substituted cyclic enones.

**2.2.2 Reaction with organoaluminum reagents**: Hoveyda and co-workers [60] investigated the NHC–copper-catalyzed asymmetric conjugate addition of alkyl- and arylaluminium reagents to five-, six-, and seven-membered β-substituted cyclic enones (Scheme 42). It is obvious that the NHC–Cu(I) complexes are generated in situ through transmetallation.

For arylation reactions, Me_2_(Ar)Al reagents were used. The substrates having a variety of substituents (R = CH_2_Bn, *n*-Bu, Me, C≡C-*n*-hep, Ph, CO_2_Me) and trialkylaluminum (alkyl = Me, Et, iBu) were used for the reactions. This approach is particularly attractive for asymmetric conjugate additions to pentenones, which are otherwise difficult to accomplish.

**Scheme 43:**
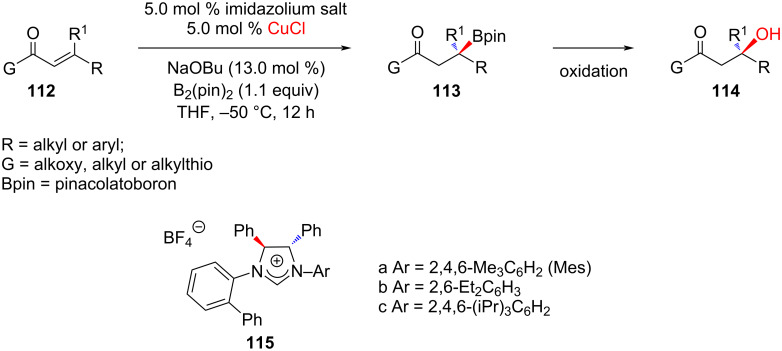
Conjugate addition of boronates to acyclic α,β-unsaturated carboxylic esters, ketones, and thioesters catalyzed by NHC–Cu(I) complex derived from **115**.

**2.2.3 Reaction with organoboron reagents:** In 2010, Hoveyda and co-workers [61] extended the application of NHC–copper catalysts to the conjugate addition of boronates to acyclic α,β-unsaturated carboxylic esters, ketones, and thioesters leading to the enantioselective formation of boron-substituted quaternary carbon stereogenic centers (Scheme 43). All transformations were accomplished by using 5 mol % of a chiral monodentate NHC–Cu complex derived from the readily available *C*_1_-symmetric imidazolium salt **115** and employing commercially available bis(pinacolato)diboron, B_2_(pin)_2_. The desired β-borylcarbonyls were obtained in 60–98% yield and >98:2 er.

Following a similar approach, Sawamura, Ohmiya and co-worker [62] accomplished the enantioselective conjugate addition of alkylboranes to α,β-unsaturated ketones in the presence of NHC–Cu(I) catalyst generated in situ from a chiral imidazolium salt and PhOK. A variety of functional groups are tolerated in the substrates and alkylboranes.

**Scheme 44:**
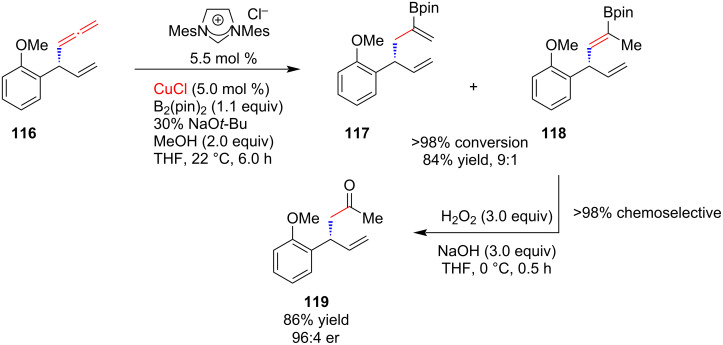
NHC–Cu(I)-catalyzed hydroboration of an allene reported by Hoveyda [63].

Later in 2012, Hoveyda and co-worker [63] used chiral bidentate NHC–Cu complexes bearing sulfonates, which was critical to regioselectivity and resulted in high selectivity for the γ-position. Thus, allenyl-containing products were generated in up to 95% yield and 99:1 er. The site-selective NHC–Cu-catalyzed hydroboration of enantiomerically enriched allenes and conversion to the corresponding β-vinyl ketones demonstrates the importance of the strategy. An example is shown below (Scheme 44).

**2.2.4 Reaction with organozinc reagents:** Organozinc reagents, such as diethylzinc, have also been employed in conjugate addition reactions. These reagents are less reactive than organolithium and Grignard reagents but can still add to a range of α,β-unsaturated carbonyl compounds, including enones, acrylates, and imines.

**Scheme 45:**
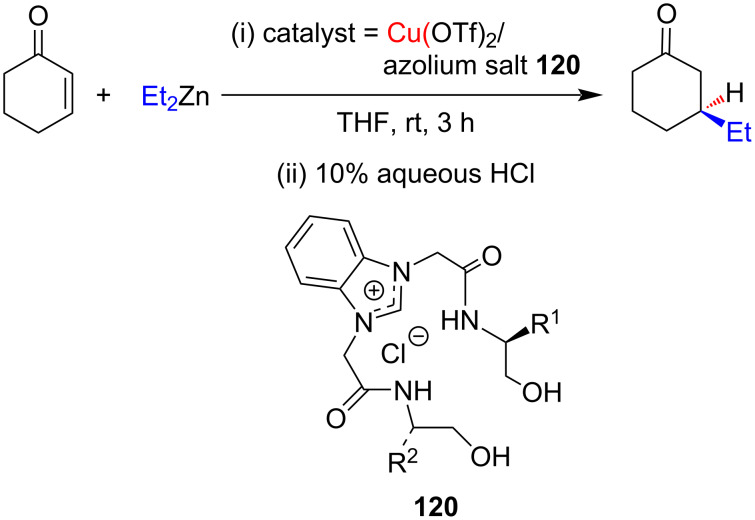
Conjugate addition of Et_2_Zn to cyclohexenone catalyzed by NHC–Cu(I) complex derived from benzimidazolium salt **120** and Cu(OTf)_2_.

In 2011 Sakaguchi and co-worker [64] accomplished the conjugate addition of Et_2_Zn to cyclohexenone employing NHC–Cu(I) complex as catalyst. After trying a number of copper salts and benzimidazolium salts, the combination of Cu(OTf)_2_ with the salt **120** (R^1^ = R^2^ = *t*-Bu) was found to give the best results with 96% yield and 63% ee (Scheme 45).

**Scheme 46:**
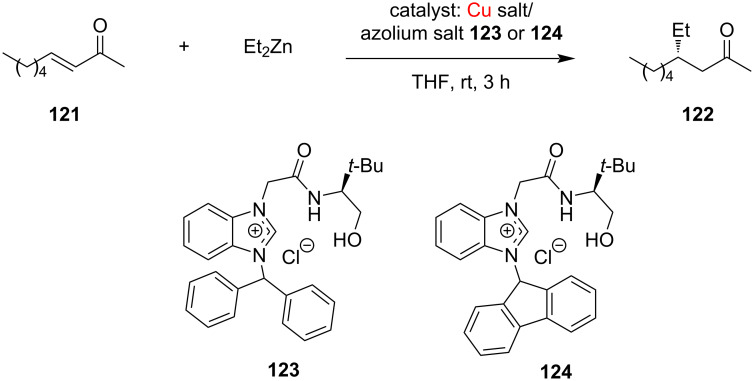
Asymmetric conjugate addition of diethylzinc to 3-nonen-2-one catalyzed by NHC–Cu complexes derived from benzimidazolium salts **123**, **124** and copper salts.

Sakaguchi and co-workers [65] further extended this work and synthesized a series of highly tunable functionalized NHC-precursor azolium salts. The efficacy of the combination of these salts with Cu salts as catalyst was investigated for the asymmetric conjugate addition (ACA) of dialkylzinc to acyclic enones and it was found that the use of an azolium salt with a sterically bulky alkyl substituent such as *N*-CHRR (e.g., NCHPh_2_ in **123**) on the azolium ring results in a marked increase in the enantioselectivity of the ACA reaction. Thus, a new functionalized azolium salt **123** was prepared and used in combination with Cu(OTf)_2_ as catalyst when products were obtained with excellent enantioselectivity (92% ee) (Scheme 46).

#### 2.3 [3 + 2] Cycloaddition reactions

**Scheme 47:**
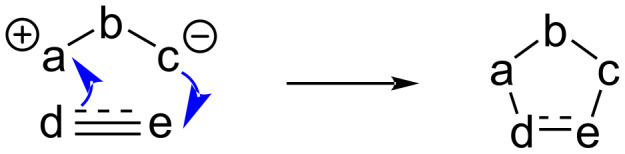
General scheme of a [3 + 2] cycloaddition reaction.

In a [3 + 2] cycloaddition reaction, a three atoms dipolar moiety (1,3-dipole) adds across two atoms of an alkene or alkyne (1,3-dipolarophile) (Scheme 47). It is also known as 1,3-dipolar cycloaddition and belongs to the general category of [_π_4_s_ + _π_2_s_] cycloadditions. It is an important method to construct a five-membered heterocyclic ring [66].

As discussed earlier, the Cu atom in NHC–Cu(I) complexes has nucleophilic character, which allows binding to the positive end of the 1,3-dipole enhancing its nucleophilicy. The latter subsequently reacts with the 1,3-dipolarophile to afford the cycloadduct.

**Scheme 48:**
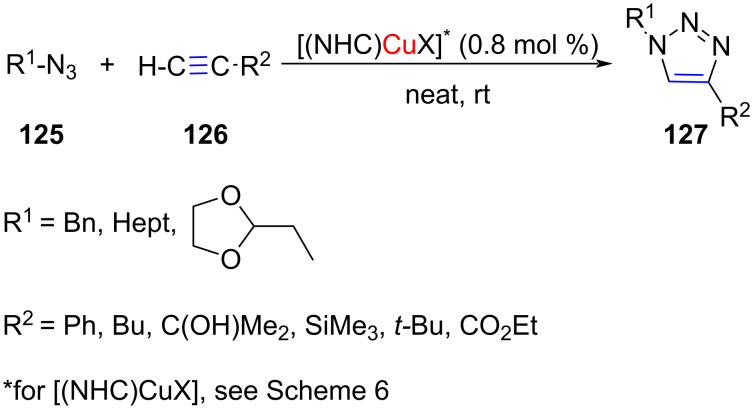
[3 + 2] Cycloaddition of azides with alkynes catalyzed by NHC–Cu(I) complexes reported by Diez-González et al. [15].

In 2010, Diez-González et al*.* utilized NHC–Cu(I) complexes, [(IAd)CuI] and [(SIMes)CuBr] (see Scheme 6 for abbreviations) for catalyzing the [3 + 2] cycloaddition of azides with alkynes under click chemistry conditions (Scheme 48) [15]. The products were obtained in high yields (93–99%).

Cazin and co-workers systematically investigated the [3 + 2] cycloaddition of a series of six azides with seven terminal alkynes catalyzed by different 1,2,3-triazolylidene–CuCl complexes. Two complexes, **61** and **65** (see Scheme 21), were found equally efficient. The products were obtained in 95 to 99% yield and the range of functionalities tolerated included nitro, nitrile, ether, carbonyl, alcohol, and amine [35].

**Scheme 49:**
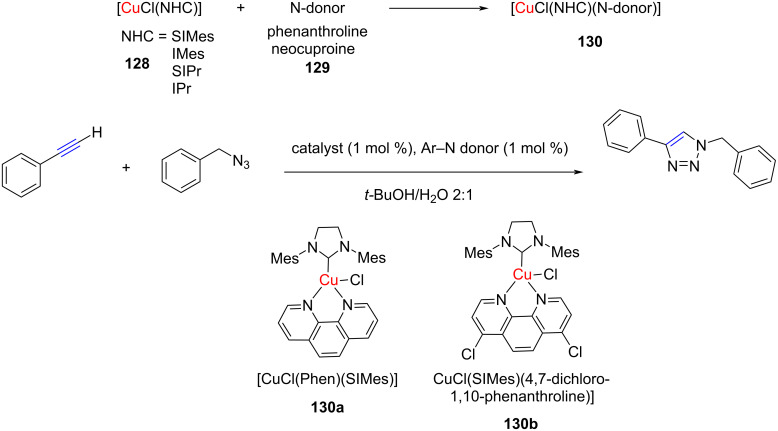
Application of NHC–CuCl/N-donor combination to catalyze the [3 + 2] cycloaddition of benzyl azide with phenylacetylene.

Gautier and co-workers studied the effect of the addition of aromatic N-donors on the catalytic activity of NHC–Cu(I) complexes for azide–alkyne [3 + 2] cycloaddition reactions [67]. They determined binding constants of four NHC–CuCl complexes with two N-donors, which revealed that addition of phenanthroline to the NHC–CuCl enhanced the catalytic activity manifold. In fact, on using [(SIMes)CuCl] with 1 mol % of phenanthroline for the [3 + 2] cycloaddition of benzyl azide with phenylacetylene, the yield of the product was 78% as against 10% in the absence of the N-donor (Scheme 49). Overall, two catalytic combinations **130a**,**b** were found to give the best results.

**Scheme 50:**
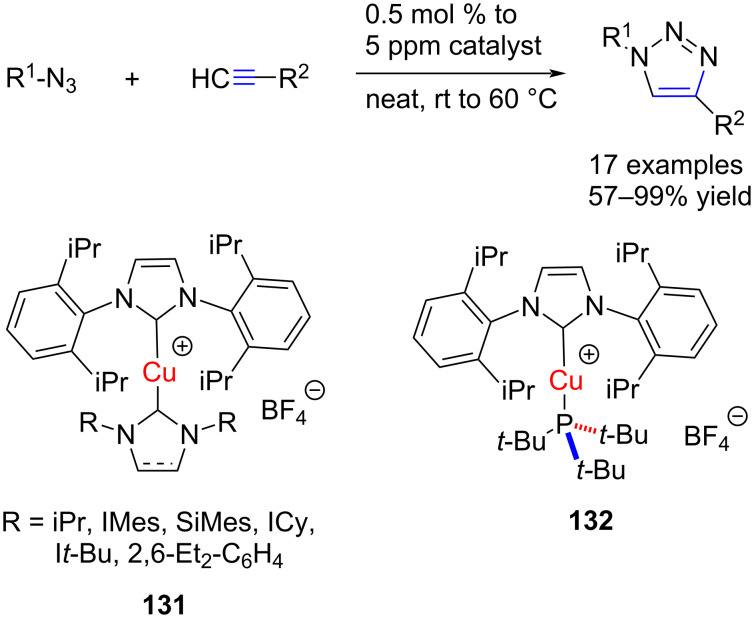
[3 + 2] Cycloaddition of azides with acetylenes catalyzed by bis(NHC)–Cu complex **131** and mixed NHC–Cu–phosphine complex **132** reported by Cazin et al. [68].

Cazin and co-workers [68] developed a new series of heteroleptic bis(NHC)–Cu(I) complexes and a mixed NHC–Cu–phosphine complex and employed these complexes as catalysts for azide–alkyne [3 + 2] cycloaddition (Scheme 50). These cationic heteroleptic bis(NHC)–Cu complexes **131** are highly active for this reaction. Furthermore, [(IPr)Cu(P*t*-Bu)_3_]BF_4_ (**132**) at only 0.5 mol % loading turned out to be a catalytic system faster than all heteroleptic bis(NHC)–Cu complexes.

**Figure 7:**
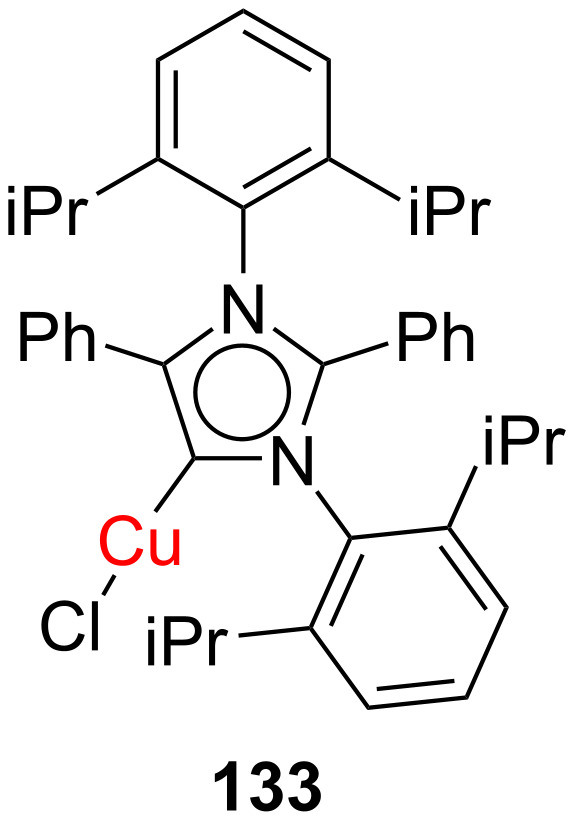
NHC–CuCl complex **133** as catalyst for the [3 + 2] cycloaddition of alkynes with azides at room temperature reported by Mandal and co-workers [69].

In 2013, Mandal and co-workers [69] prepared the NHC–CuCl complex **133** (Figure 7) through deprotonation of the corresponding imidazolium salt with KN(SiMe_3_)_2_ and used it successfully for catalyzing the [3 + 2] cycloaddition of alkynes with azides. The reaction of benzyl azide with phenylacetylene could be accomplished with only 0.005 mol % catalyst loading giving a quantitative yield of the product at rt.

**Scheme 51:**
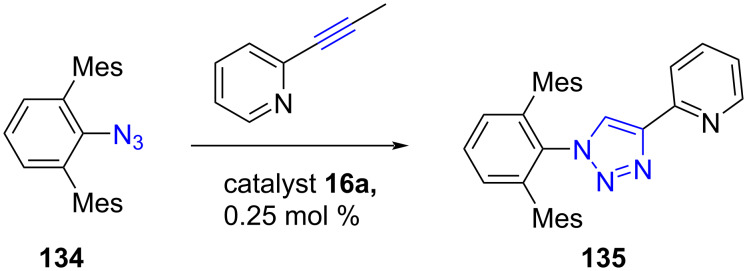
[3 + 2] Cycloaddition of a bulky azide with an alkynylpyridine using [(NHC)Cu(μ-I)_2_Cu(NHC)] copper catalyst reported by Sarkar [22].

As discussed earlier, Sarkar and co-worker [22] synthesized [(NHC)Cu(μ-I)_2_Cu(NHC)] complexes **16** (Scheme 8) from CuI and benzimidazolium salts and **18** (Scheme 9) from 1,2,3-triazolium salts. Both types of complexes turned out to be highly efficient catalysts for the [3 + 2] cycloaddition of azides with alkynes. The reactions were complete in short times and required low catalyst loadings and a single product was obtained in each case. Furthermore, the cycloaddition with an internal alkyne could also be achieved using these catalysts. Interestingly, the catalysts were found effective in the cycloaddition of 2-(prop-1-yn-1-yl)pyridine with bulky azide **134** to afford 1-(2,6-dimesityl)phenyl-4-(2-pyridyl)-1,2,3-triazole (**135**), which is otherwise difficult to achieve (Scheme 51).

**Scheme 52:**
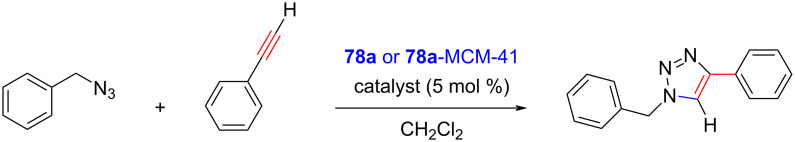
[3 + 2] Cycloaddition of benzyl azide with phenylacetylene under homogeneous and heterogeneous catalytic conditions reported by Oro et al [39].

Oro and co-workers employed complexes **78a** and **78a-MCH-41** (Scheme 27) as homogeneous and heterogeneous catalysts, respectively, for the [3 + 2] cycloaddition of benzyl azide with phenylacetylene (Scheme 52) [39]. In contrast to the hydrosilylation reaction (see section 2.1), both complexes catalyzed the cycloaddition reaction; however, the heterogeneous catalyst was found to be less active than the homogeneous catalyst.

**Scheme 53:**
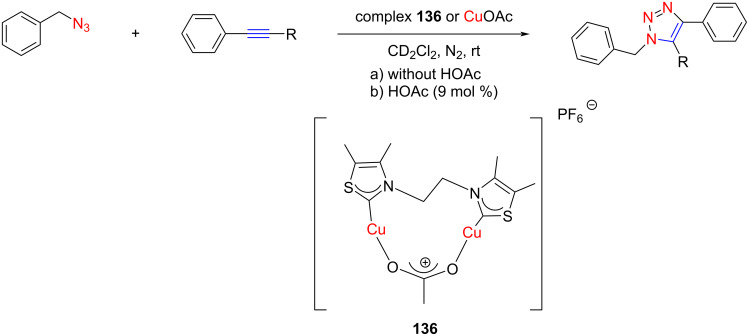
[3 + 2] Cycloaddition of benzyl azide with acetylenes catalyzed by bisthiazolylidene dicopper(I) complex **136** reported by Straub and co-workers [70].

Straub and co-workers [70] in 2016 instead explored the application of a thiazolylidene-based Cu(I) complex as catalyst for [3 + 2] cycloaddition reactions. They prepared an ethylene-linked bisthiazol-2-ylidene dicopper(I) complex **136** which showed high catalytic activity (Scheme 53). The activity increased upon addition of acetic acid, particularly for more acidic alkyne substrates. In contrast to the reaction under homogeneous catalysis conditions by a saturated solution of Cu(OAc), the reaction in the presence of catalyst **136** was 4.5 times (in the absence of HOAc) and 7.5 times (with HOAc) faster.

**Figure 8:**
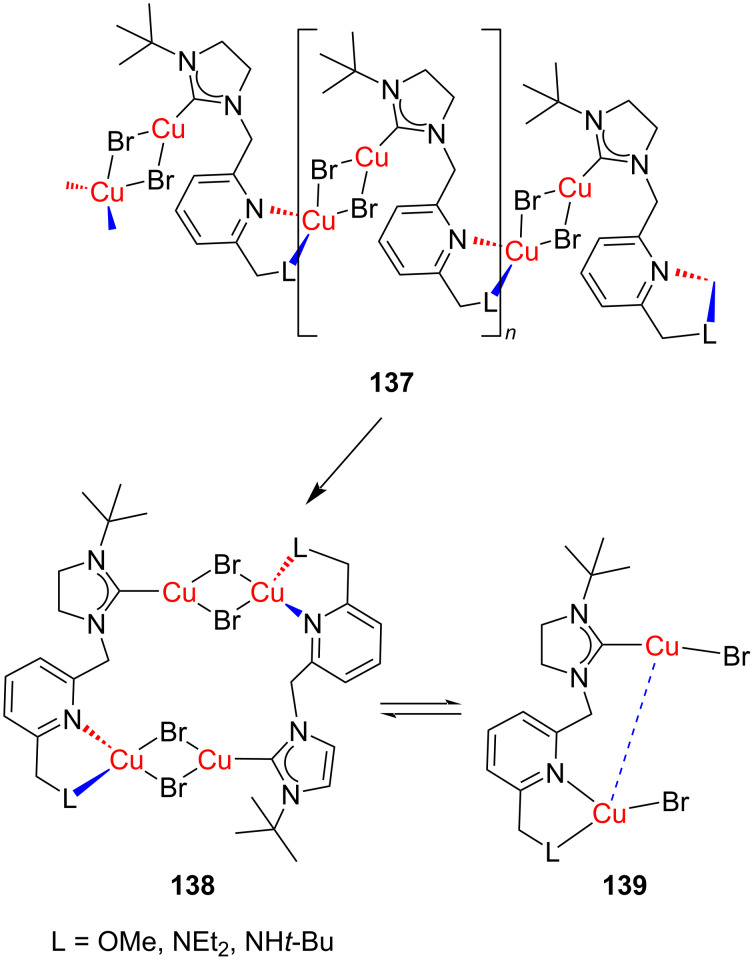
Copper (I)–NHC linear coordination polymer **137** and its conversion into tetranuclear (**138**) and dinuclear (**139**) species reported by Pérez-Torrente and co-workers [71].

Recently in 2022, Pérez-Torrente and co-workers [71] reported the preparation of a polymeric linearly coordinated Cu(I)–NHC compound **137** composed of bimetallic [Cu(μBr)_2_] units linked through a lutidine-based NHC–py–NEt_2_ ligand (Figure 8). The polymeric complex was assumed to break down during the reaction in solution to generate a tetranuclear [Cu_2_(μBr)_2_(*t*-BuImCH_2_pyCH_2_L)]_2_ species **138** acting similar to the NHCs. A very low loading of these complexes was sufficient to catalyze the azide–alkyne cycloaddition. A theoretical investigation at the DFT level confirmed the participation of the dinuclear species [(CuBr)_2_(μ-*t*-BuImCH_2_pyCH_2_NEt_2_)] **139**, generated from the tetranuclear complex, as the catalytically active species.

#### 2.4 A^3^ reactions

**Scheme 54:**
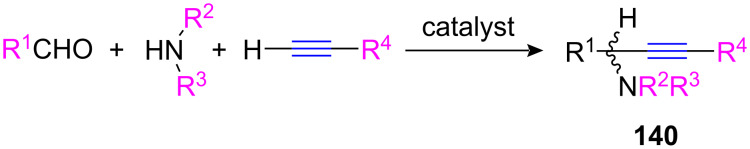
An A^3^ reaction.

In recent years, the three component reaction of an aldehyde, a secondary amine and a terminal alkyne, known as A^3^ reaction to afford chiral propargylamines **140** has received much attention. The latter compounds have been found versatile synthons for medicinally important and naturally occurring nitrogen-containing compounds (Scheme 54) [72–74].

In the past, several transition-metal catalysts were used for this reaction and Li and co-workers reviewed the use of transition-metal salts for the A^3^ reactions [75]. However, the major drawback with using these catalysts in A^3^ reactions was the loss of the catalyst at the end of the reaction. Furthermore, on using Au(I), Ag(I), and Cu(I) in ionic liquids, as well as supported Au(III), Ag(I), CuI, and CuCl to catalyze A^3^ coupling reactions under heterogeneous conditions, although the transition-metal catalysts were preserved, the reactions require high temperature conditions [76].

**Scheme 55:**
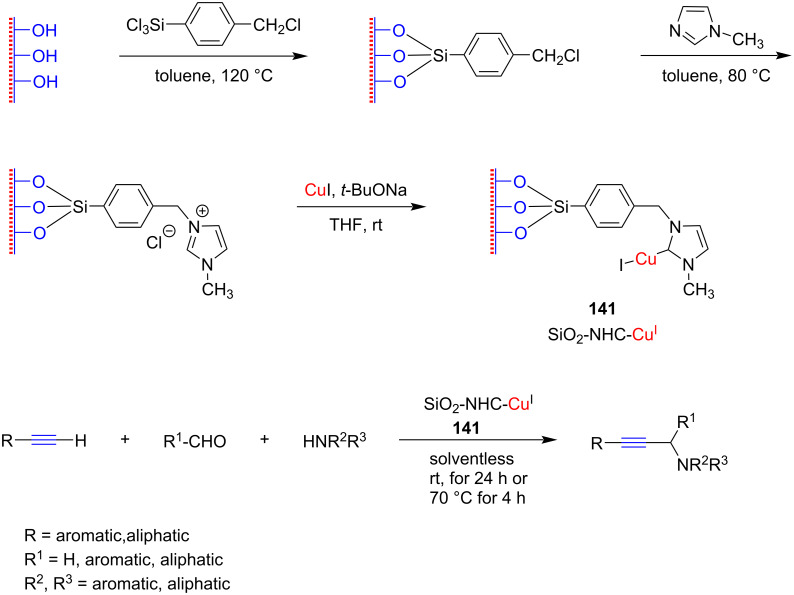
Synthesis of SiO_2_-immobilized NHC–Cu(I) catalyst **141** and its application in the A^3^-coupling reaction reported by Wang and co-workers [77].

In 2008, Wang and co-workers [77] for the first time employed an NHC–Cu(I) complex (2 mol %) and its silica-immobilized version **141** (2 mol %) as catalyst for an A^3^ coupling reaction (Scheme 55). A wide range of combinations of aldehydes, alkynes, and secondary amines including aromatic as well as aliphatic substrates were used and the products were obtained in up to 95% yield. The effects of solvents and temperature were also investigated. Polar solvents such as acetone or dichloromethane increased the reactivity, resulting in quantitative conversion, but toluene, acetonitrile, and tetrahydrofuran (THF), which had previously been reported to be extremely effective in the presence of copper salts, gave poor yields.

In 2013, Navarro and co-worker [78] used [(NHC)Cu(I)]X (X = Cl, NHC = IPr, SIPr, IMes, and SIMes) complexes as catalysts for the A^3^ reaction. The N-substituent in the NHC fragment had a considerable effect on the results with IPr and its saturated equivalent (SIPr) performed the best (82 and 94% conversion, respectively), while IMes and SIMes showed minimal activity (4 and 7% conversion). On the other hand, the saturation of the NHC backbone had just a little influence. Furthermore, replacing chloride with iodide resulted in a relatively small decrease in reactivity.

**Scheme 56:**
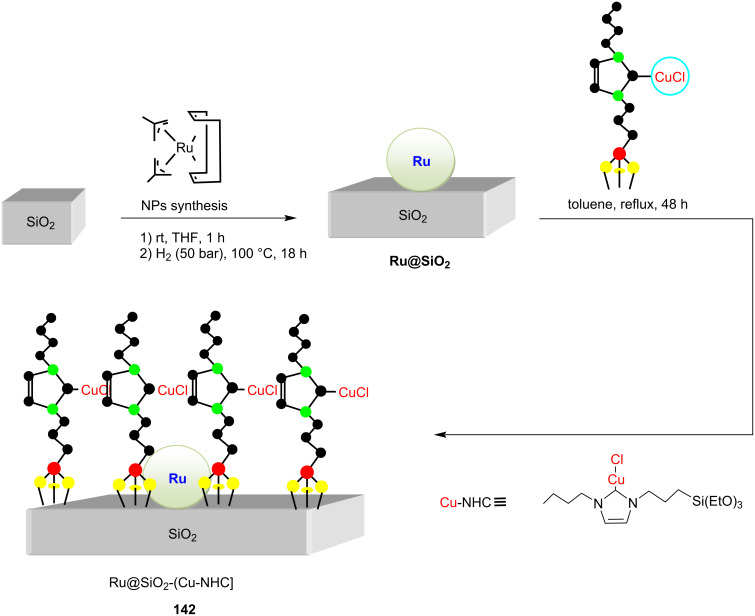
Preparation of dual-purpose Ru@SiO_2_–[(NHC)CuCl] catalyst system **142** developed by Bordet, Leitner and co-workers [79].

**Scheme 57:**
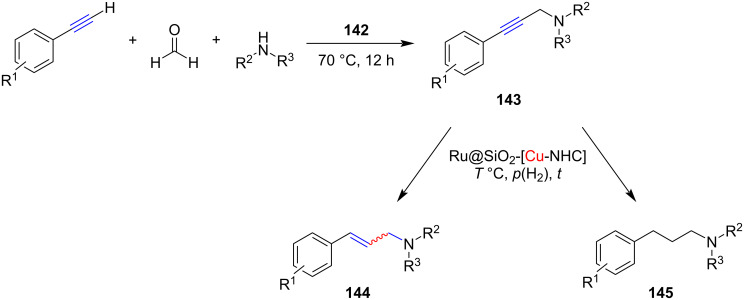
Application of the catalyst system Ru@SiO_2_–[Cu(NHC)] **142** to the one-pot tandem A^3^ reaction and hydrogenation.

In an interesting research paper, Bordet, Leitner and co-workers [79] reported the development of a multifunctional catalytic system **142** incorporating ruthenium nanoparticles (RuNPS) and an NHC–Cu–Cl complex supported on silica (Scheme 56). The catalyst, Ru@SiO_2_–[Cu(NHC)] was successfully applied to a one-pot tandem A^3^ reaction of an aldehyde, alkyne, and secondary amine followed by hydrogenation of the resulting propargylamines to give allylamines **144** and alkylamines **145** in quantitative yields (Scheme 57). This system is made up of a silica support that has been modified with a covalently linked NHC–Cu complex for the A^3^ coupling, as well as RuNPs for hydrogenation. Detailed investigations revealed that the A^3^ reaction is catalyzed by the NHC–Cu complex while the selective hydrogenation is catalyzed by Ru(0) nanoparticles.

The versatility of this tandem catalytic approach was established by using a wide range of substrates. Furthermore, the catalytic activity and selectivity remained fairly constant for three cycles, with a slight lowering of the hydrogenation activity with time.

**Scheme 58:**
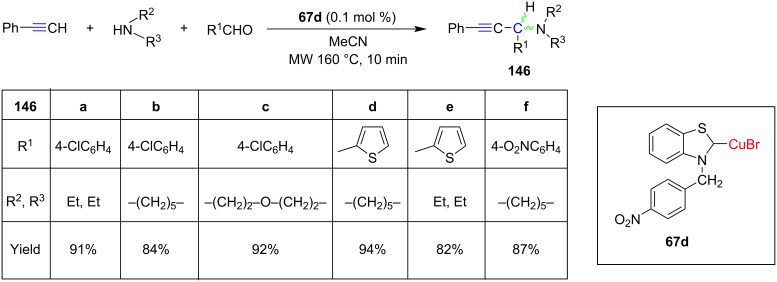
A^3^ reaction of phenylacetylene with secondary amines and aldehydes catalyzed by benzothiazolylidene–CuBr complex **67d**.

Our group for the first time used the NHC benzothiazolylidene–CuBr complex **67d** (Scheme 22) for catalyzing A^3^ reactions under microwave conditions. The products **146** were obtained in high yields (Scheme 58) [36].

**Figure 9:**
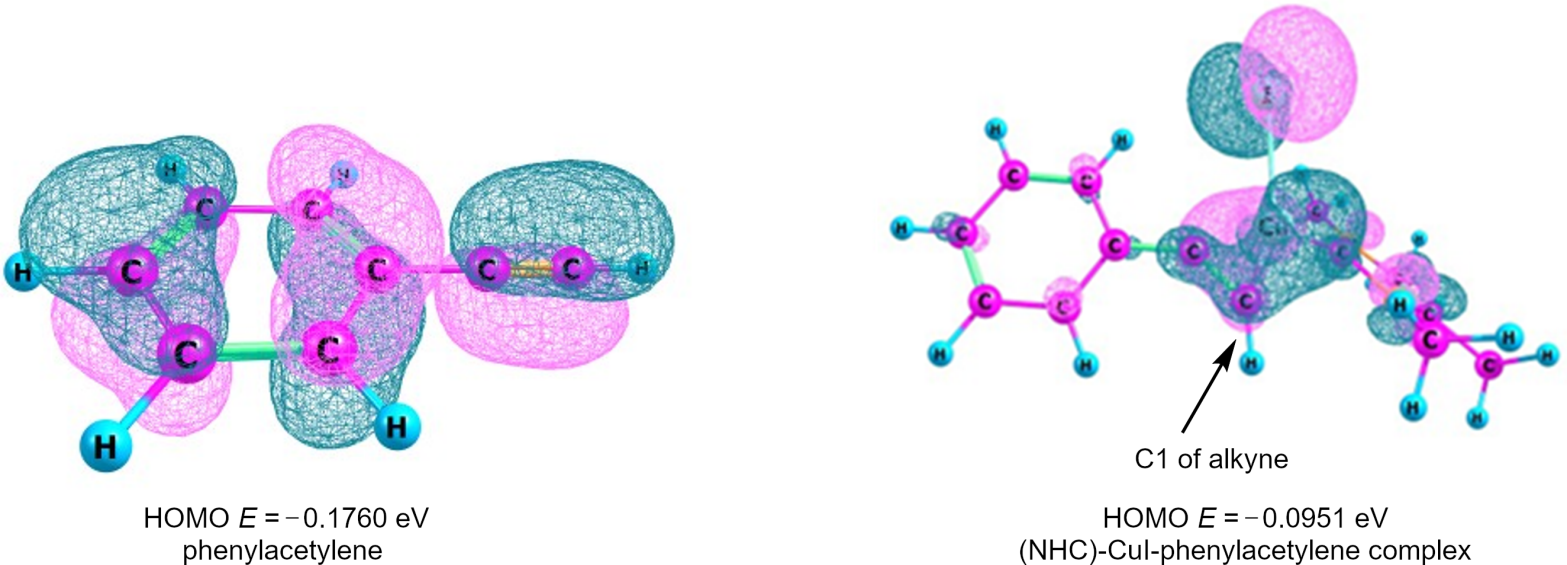
Kohn–Sham HOMOs of phenylacetylene and NHC–Cu(I)–phenylacetylene complex computed at the B3LYP/def2-SVP level of theory.

**Figure 10:**
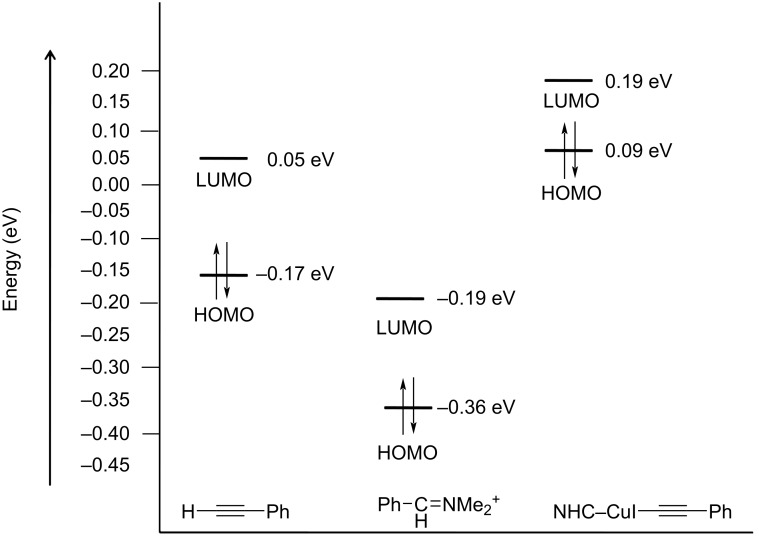
Energies of the FMOs of phenylacetylene, iminium ion, and NHC–Cu(I)–phenylacetylene complex computed at the B3LYP/def2-SVP level of theory.

The role of NHC–Cu(I) in catalyzing the A^3^ reaction was investigated theoretically also by computing a model reaction at the B3LYP/def2SVP level of theory [36]. In contrast to the HOMO of phenylacetylene which is distributed over the whole molecule, the HOMO of NHC–Cu(I)-complexed phenylacetylene is centered on C1 enhancing its nuceophilicity (Figure 9). The Mulliken charge, obtained from the NBO calculations, was found to be almost double at the 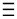
C–H atom in the complexed alkyne (−0.294) as compared to the free alkyne (−0.129). Furthermore, as depicted in Figure 10, the HOMO–LUMO energy gap in the NHC–Cu(I)–alkyne complex becomes much smaller than in the uncomplexed alkyne.

#### 2.5 Boration and hydroboration

NHC–Cu(I) complexes have also been successfully applied to catalyze the boration and hydroboration of carbonyl compounds, allenes, and similar substrates to obtain boronated products [80].

**Scheme 59:**
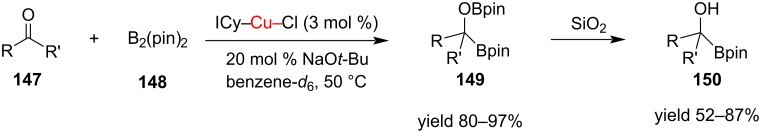
NHC–Cu(I) catalyzed diboration of ketones **147** by reacting with bis(pinacolato)diboron (**148**) reported by Clark and co-workers [81].

Clark and co-workers [81] accomplished the diboration of several functionalized ketones catalyzed by Icy–Cu–O*t*-Bu to obtain α-hydroxyboronate esters (Scheme 59). The catalyst freshly generated in situ showed greater activity and the products were obtained in moderate to high yields. Furthermore, a high diastereoselectivity (dr > 99:1) was observed in the reaction of chiral ketones.

**Scheme 60:**
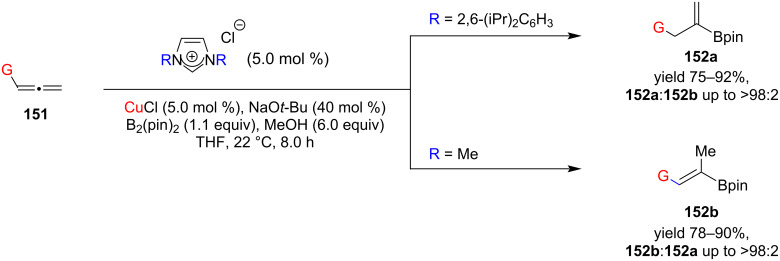
Protoboration of terminal allenes catalyzed by NHC–Cu(I) complexes reported by Hoveyda and co-workers [82].

Hoveyda and co-workers [82] employed two types of NHC–Cu(I) complexes to catalyze the protoboration of terminal allenes to obtain vinylboranes (Scheme 60). A set of alkyl- and aryl-substituted allenes was used as substrates. The transformations of sterically congested substrates, including those carrying a quaternary center not only proceeded efficiently but also were more selective. The NHCs with larger aryl units deliver higher selectivity ((Z/E: >98:2).

Cazin and co-workers [83] instead used [IMes–CuCl] as catalyst for the borylation of internal alkynes with bis(pinacolato)diboron to obtain vinylboranes. The reaction could be performed in air.

**Scheme 61:**
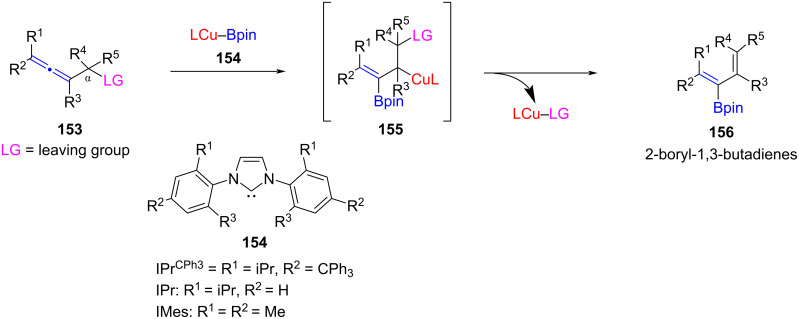
NHC–CuCl-catalyzed borylation of α-alkoxyallenes to give 2-boryl-1,3-butadienes.

Tsuji and co-workers [84] prepared 2-boryl-substituted 1,3-butadienes, which are otherwise difficult to synthesize, through NHC–CuCl-catalyzed borylation of α-alkoxyallenes with B_2_(pin)_2_ (Scheme 61). Out of the three ligands, **154** the one incorporating a bulky trityl (–CPh_3)_ group was found to be most effective giving the product in up to 99% yield. A low loading (2 mol %) of the catalyst was needed and the reactions occurred at rt.

**Scheme 62:**
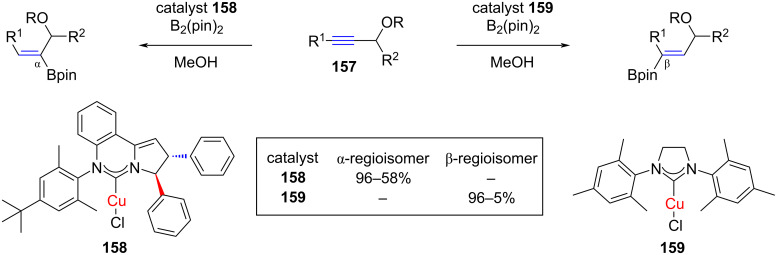
Regioselective hydroborylation of propargylic alcohols and ethers catalyzed by NHC–CuCl complexes **158** and **159** reported by McQuade and co-workers [85].

McQuade and co-workers observed that the regioselectivity of the NHC–CuCl-catalyzed hydroborylation of propargylic alcohols (**157**, R = H) and ethers (**157**, R = aryl, alkyl) was dependent on the size of the NHC [85] (Scheme 62). Interestingly, the application of the catalyst **158** comprising a bulkier NHC preponderantly led to the sterically crowded α-regioisomer, while the use of the less congested catalyst **159**, gave preferentially the β-regioisomer. The reaction tolerates a wide range of functional groups.

**Scheme 63:**
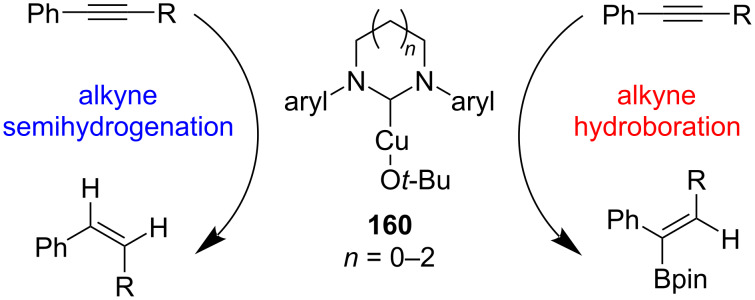
NHC–CuO*t*-Bu-catalyzed semihydrogenation and hydroborylation of alkynes.

Whittlesey and co-workers [86] studied the effects of differently sized NHCs on the NHC–CuO*t*-Bu-catalyzed semihydrogenation and hydroborylation of alkynes with silanes/*t*-BuOH and HBP, respectively. The Cu(I) complexes of five-, six-, and seven-membered NHCs were prepared through protonolysis of NHC–CuMes with *t*-BuOH (Scheme 63). Increasing the size of the heterocyclic ring was accompanied by higher selectivity to give mainly (*Z*)-alkenes and α-hydroboration products. Furthermore, the presence of 2,6-dimethylphenyl-derived N-substituents on the NHC were optimal for the catalysis.

**Scheme 64:**
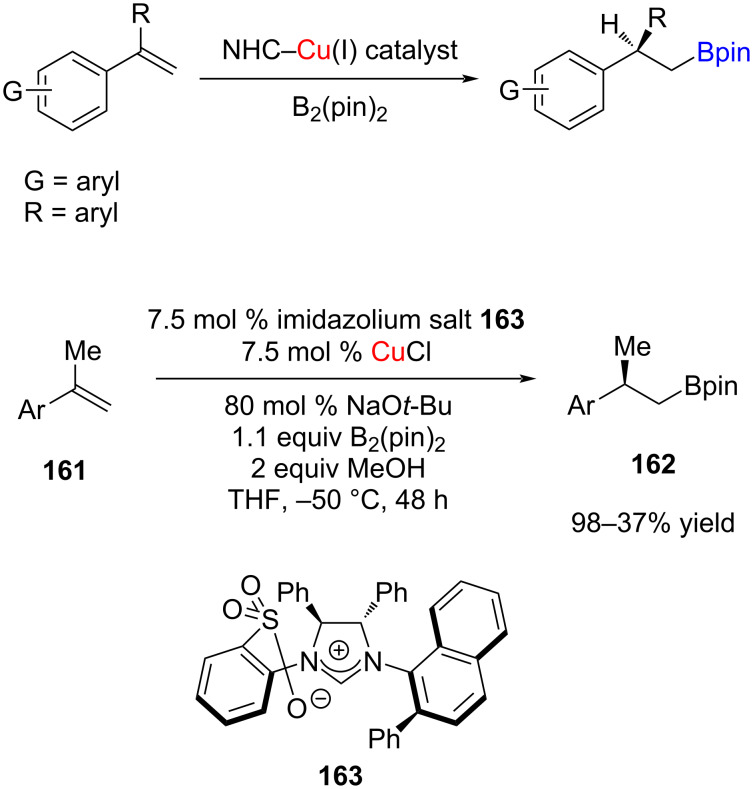
Enantioselective NHC–Cu(I)-catalyzed hydroborations of 1,1-disubstituted aryl olefins reported by Hoveyda et al. [87].

Hoveyda and co-workers [87] reported the NHC–Cu(I)-catalyzed site- and enantioselective hydroboration of 1,1-disubstituted aryl olefins to obtain α-alkyl-β-pinacolatoboranes with >98% site-selectivity, in up to 98% yield and er = 96.5:3.5. Reactions involved a wide range of acyclic 1,1-disubstituted aryl olefins. Several *C*_1_-symmetric bidentate ligands derived from enantiomerically pure imidazolinium salts were examined for their efficiency. For example, a remarkable improvement in the enantiomeric purity of the product (>98% conv., 85.5:14.5 er) was achieved while using the naphthyl-substituted salt **163** (Scheme 64).

**Scheme 65:**
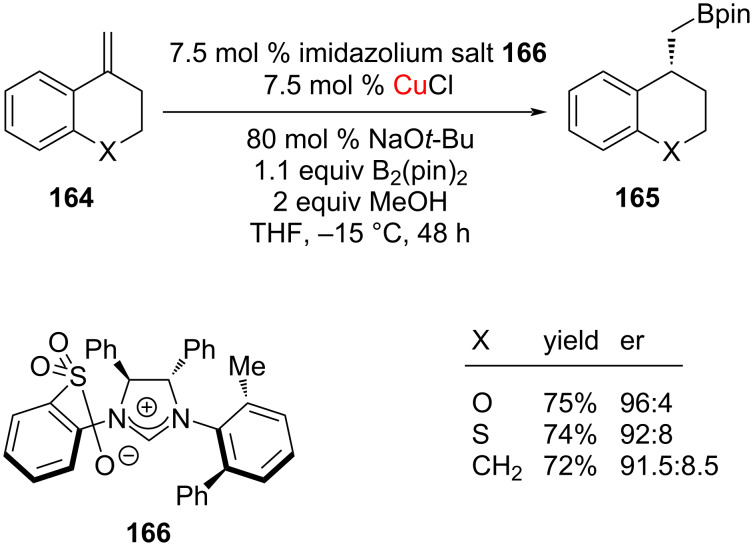
Enantioselective NHC–Cu(I)-catalyzed hydroboration of exocyclic 1,1-disubstituted alkenes reported by Hoveyda et al. [87].

Besides, a highly enantioselective catalytic hydroboration could be achieved from several exocyclic 1,1-disubstituted alkenes **164**; in this transformation, the Cu complex derived from salt **166** was found to be most effective (Scheme 65) [87].

**Scheme 66:**
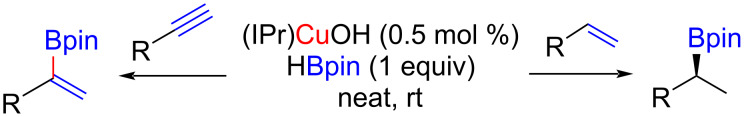
Markovnikov**-s**elective NHC–CuOH-catalyzed hydroboration of alkenes and alkynes reported by Jones et al. [88].

Jones and co-workers [88] developed a highly Markovnikov-selective hydroboration of alkenes and alkynes catalyzed by NHC–CuOH (Scheme 66). The products were obtained in high yields with low (0.5 mol %) catalyst loading. Substrates with electron-withdrawing groups are tolerated, whereas strong electron-releasing groups decrease the reactivity. Steric hindrance also plays a crucial role, with *ortho*-substitution resulting in reduced catalytic activity.

**Scheme 67:**
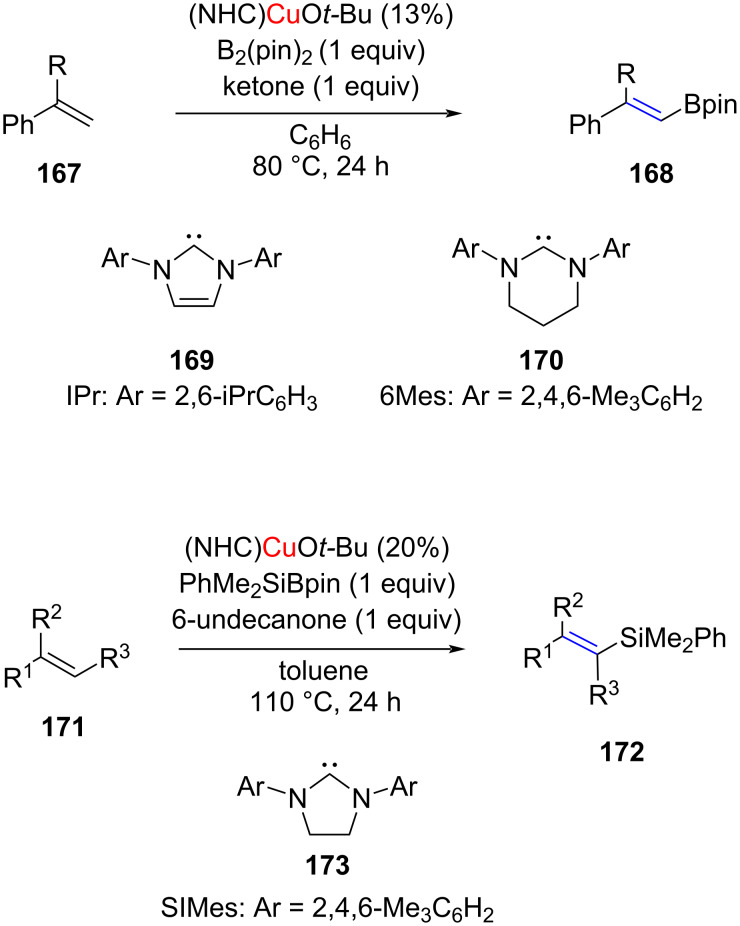
Dehydrogenative borylation and silylation of styrenes catalyzed by NHC–CuO*t*-Bu complexes developed by Mankad and co-worker [89].

Mankad and co-worker [89] developed dehydrogenative borylation and silylation of styrenes catalyzed by NHC–CuO*t*-Bu to afford vinylboronates **168** and vinylsilanes **172** including trisubstituted derivatives, which are otherwise difficult to obtain through alkyne hydrofunctionalization. For the dehydrogenative borylation, two types of NHCs, **169** and **170**, were employed and the use of a ketone was necessary to induce C–H functionalization selectivity in preference to C=C functionalization. The products were obtained in moderate to high yields (Scheme 67).

#### 2.6 N–H and C(sp^2^)–H carboxylation

**Scheme 68:**
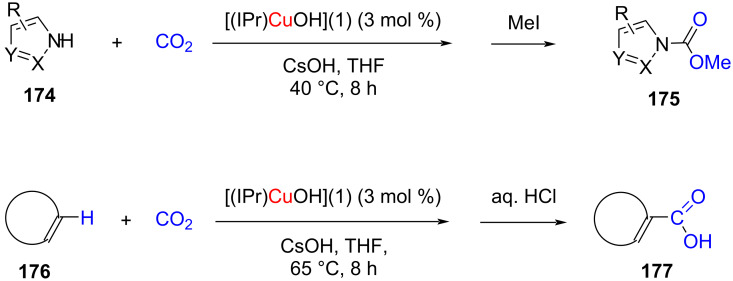
N–H/C(sp^2^)–H carboxylation catalyzed by NHC–CuOH complexes.

The application of the [(IPr)CuOH] complex as catalyst for the N–H/C(sp^2^)–H carboxylation of acidic arenes and heteroarenes has been reported by Cazin, Nolan and co-workers [90]. In this context, it was found that a combination of [(IPr)CuOH] (3 mol %) with CsOH in THF afforded the best results (Scheme 68), whereas the SIPr, IMes, and SIMes-based NHC–Cu complexes were found to be less effective. This method afforded the products with high selectivity and it could be extended to a variety of substrates, such as benzoxazole, benzothiazole, oxazole, and even acidic hydrocarbons and aniline.

**Scheme 69:**
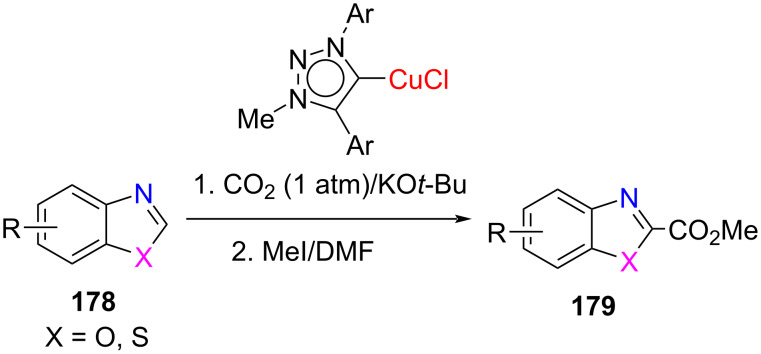
C–H Carboxylation of benzoxazole and benzothiazole derivatives with CO_2_ using a 1,2,3-triazol-5-ylidene copper(I) complex.

Fukuzama and co-workers [91] accomplished the C–H carboxylation of benzoxazole and benzothiazole derivatives with CO_2_ using 1,2,3-triazol-5-ylidene copper(I) complexes (tzNHC–Cu) as the catalyst followed by treatment with alkyl iodide to obtain the corresponding esters in moderate to very good yields. The catalytic activity of (tzNHC–Cu) was found to be better than the imidazol-2-ylidene copper(I) complex, [(IPr)CuCl] (Scheme 69).

**Scheme 70:**
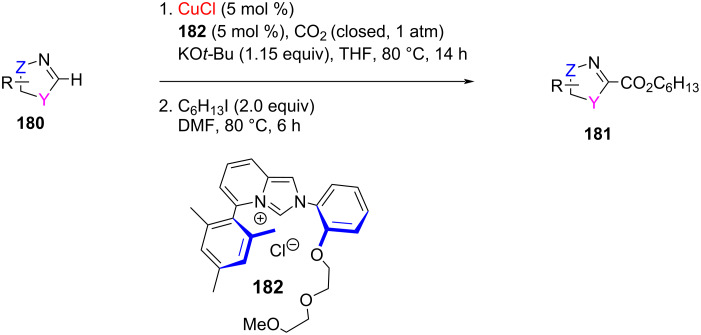
Use of Cu(I) complex derived from diethylene glycol-functionalized imidazo[1,5,*a*] pyridin-3-ylidenes (DEG-ImPy) as catalyst for C–H carboxylation of heterocyclic substrates with CO_2_.

Hong and co-workers [92] developed diethylene glycol-functionalized imidazo[1,5,*a*]pyridin-3-ylidenes (DEG-ImPy) as a bifunctional NHC ligand. The Cu catalyst generated in situ with the DEG-ImPy·HCl salt **182** efficiently catalyzed direct C–H carboxylation of various heterocyclic compounds with CO_2_ resulting in higher yields than those obtained with the imidazolylidene carbene ligand, IPr (Scheme 70).

#### 2.7 C(sp^2^)–H Alkenylation and allylation

**Scheme 71:**
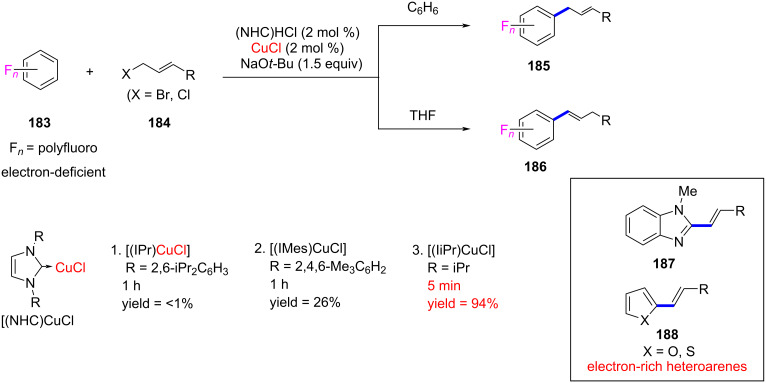
Allylation and alkenylation of polyfluoroarenes and heteroarenes catalyzed by NHC–Cu(I) complexes reported by Chang and co-worker [93].

In 2016, Chang and co-worker [93] achieved an NHCs–Cu-catalyzed efficient C(sp^2^)–H allylation of polyfluoroarenes **183** and isomerization-induced alkenylation of electron-rich heteroarenes **187** and **188** utilizing allyl halides as reactants. The same catalyst system was found to effectively promote double-bond migration of the initially formed allylarenes resulting in vinylarene products. This method has been successfully used to alkenylate a variety of electron-rich as well as electron-deficient (hetero)arenes. Of the three imidazolylidene derived NHC–CuCl catalysts, the best results (up to 94% yield) were obtained with [(IiPr)CuCl]. The method has several key features, such as mild reaction conditions, tolerance of various functionalities, applicability to a wide range of heteroarenes and allyl halides, and high stereoselectivity (Scheme 71).

**Scheme 72:**
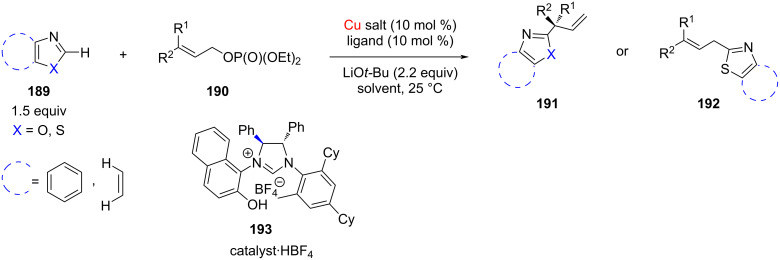
Enantioselective C(sp^2^)–H allylation of (benz)oxazoles and benzothiazoles with γ,γ-disubstituted primary allyl phosphates catalyzed by NHC–Cu(I) complexes.

The enantioselective C(sp^2^)–H allylation of (benz)oxazoles and benzothiazoles with γ,γ-disubstituted primary allyl phosphates catalyzed by NHC–Cu(I) complexes was reported by Ohmiya, Sawamura and co-workers (Scheme 72) [94]. Some of the characteristic features of this methodology are: enantioselective formation of the quaternary stereocenters, broad substrate scope, high enantioselectivity, and branched:linear selectivity. Furthermore, the NHC–Cu complex incorporating the chelating *N*-2-naphthol moiety **193** afforded the highest yield and enantioselectivity. On protecting the hydroxy group in the ligand as methyl ether, the reaction efficiency decreased remarkably. However, on using NHC ligands without oxygen atom, such as analogues of **193**, IMes, and SIMes, no conversion occurred.

#### 2.8 C(sp^2^)–H Arylation

**Scheme 73:**
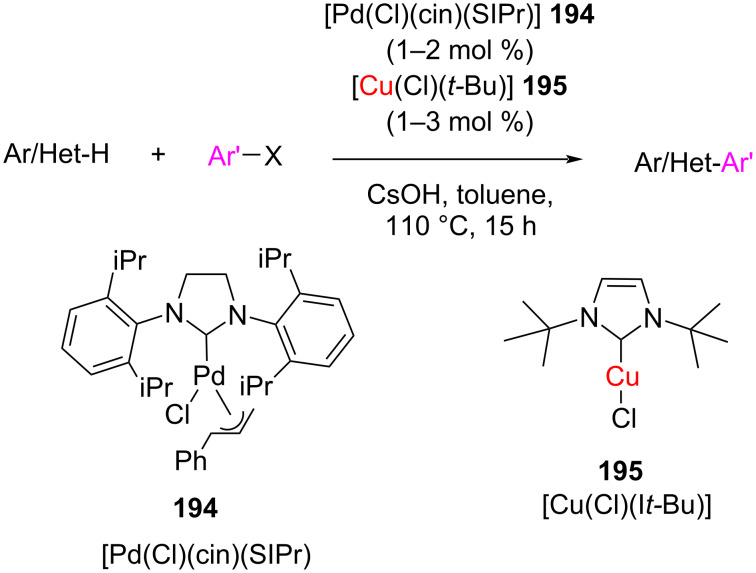
C(sp^2^)–H arylation of arenes catalyzed by dual NHC–Cu/NHC–Pd catalytic system.

In 2014, Cazin and co-workers accomplished the C(sp^2^)–H arylation of acidic arenes by using a cooperative NHC–Cu/NHC–Pd catalytic system in the presence CsOH (Scheme 73) [95]. [(I*t*-Bu)CuCl] changes in situ into the catalytically active species [(I*t*-Bu)CuOH], which induces C(sp^2^)–H activation to generate an aryl–Cu–NHC species. This is followed by the reaction with NHC–Pd to produce an Ar–Pd(NHC)Cl intermediate through the oxidative addition to Pd(0)NHC. Finally, transmetallation of [(I*t*-Bu)Cu(Ar)] with [(SIPr)Pd(Ar)Cl] followed by reductive elimination leads to biaryl product. No conversion occurred using either Pd–NHC or Cu–NHC alone.

#### 2.9 C(sp^2^)–H amidation

**Scheme 74:**
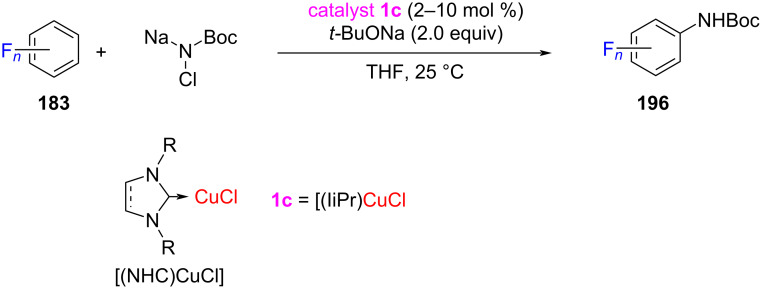
C(sp^2^)–H Amidation of (hetero)arenes with *N*-chlorocarbamates/*N*-chloro-*N*-sodiocarbamates catalyzed by NHC–Cu(I) complexes.

Chang and co-workers [96] reported an NHC–Cu-catalyzed direct amidation of C–H bonds by using *N*-chlorocarbamates or *N*-chloro-*N*-sodiocarbamates as amino source. In this mechanism, a copper–aryl intermediate reacts with the amidating reagent leading to the isolation of copper–arylcarbamato species and the desired product. The developed amidation protocol works highly efficiently and selectively over a broad range of substrates including polyfluorobenzenes, azoles, and quinoline *N*-oxides (Scheme 74).

#### 2.10 C(sp^2^)–H thiolation

**Scheme 75:**
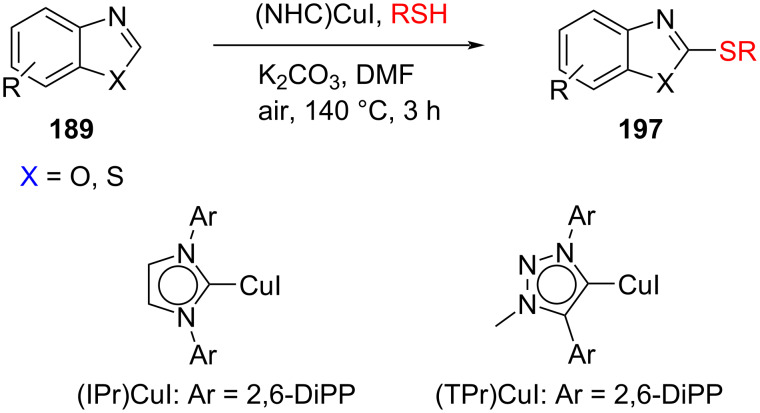
NHC–CuI catalyzed thiolation of benzothiazoles and benzoxazoles.

Fukuzawa and co-workers [97] accomplished the oxidative thiolation of benzothiazoles and benzoxazoles with aryl and alkyl thiols catalyzed by NHC–copper(I) complexes to give 2-(arylthio)- and 2-(alkylthio)benzoxazoles/-benzothiazoles in moderate to good yields. Several catalysts based on imidazol-2-ylidene and 1,2,3-triazol-5-ylidene, [(IPr)CuI[, [(IMes)CuI], [(TPr)CuI], and [(TMes)CuI] were examined. On using [(IPr)CuI] as a catalyst, the reaction was fastest and its yield reached 95% after 3 h, while with [(IMes)CuI], the reaction time was 6 h giving a maximum yield of 85%. For [(TPr)CuI] as the catalyst, the yield was 95% after 7 h; CuI did not catalyze the reaction (Scheme 75).

## Conclusion

Application of N-heterocyclic carbene–metal complexes in general and NHC–Cu(I) complexes in particular has made remarkable progress during the last decade. A variety of new NHC–Cu(I) complexes have been developed and applied as catalysts for different types of organic reactions. However, a critical survey reveals that these catalysts are based mainly on imidazol(in)ylidene skeletons and only very limited work has been done on catalysts prepared with NHCs derived from six-membered and larger rings. Also the application of NHCs derived from the thiazole nucleus is almost nil. In view of this, it may be perceived that the metal complexes from the NHCs derived from other N/S heterocycles will be developed and used as catalysts. Furthermore, it may be expected that in the coming years, the application of NHC–Cu(I) complexes will be extended to new reactions, such as oxa-Michael reactions which may lead to pharmacologically useful ethers. Structural modelling of the NHC–Cu(I) complexes for achieving higher stereoselectivity of the products can be perceived another important field of activity in future.
